# Epigenetic mechanism of *Gtl2*-miRNAs causes the primitive sheep characteristics found in purebred Merino sheep

**DOI:** 10.1186/s13578-023-01142-z

**Published:** 2023-10-13

**Authors:** Jiankui Wang, Guoying Hua, Jianfei Chen, Kai Cui, Zu Yang, Deping Han, Xue Yang, Xianggui Dong, Yuhao Ma, Ganxian Cai, Yuanyuan Zhang, Jinnan Li, Yurong Tai, Lai Da, Xinhai Li, Lina Ma, Qing Ma, Rui Li, Jianbin Liu, Hesham Y. A. Darwish, Keliang Wu, Weiheng Rong, Wansheng Liu, Yaofeng Zhao, Xuemei Deng

**Affiliations:** 1 Beijing Key Laboratory for Animal Genetic Improvement & Key Laboratory of Animal Genetics, Breeding and Reproduction, Ministry of Agriculture & State Key Laboratory of Animal Biotech Breeding, China Agricultural University, Beijing, 100193 China; 2 Feed Research Institute, Chinese Academy of Agricultural Sciences, Beijing, 100086 China; 3 Inner Mongolia Academy of Agricultural and Animal Husbandry Sciences, Huhhot, 010031 China; 4 Jinfeng Animal Husbandry Group Co., Ltd., Chifeng, 024000 China; 5 College of Agriculture, Ningxia University, Yinchuan, 750021 China; 6 Institute of Animal Science, Ningxia Academy of Agriculture and Forestry Sciences, Yinchuan, 750002 China; 7 Lanzhou Institute of Husbandry and Pharmaceutical Sciences, Chinese Academy of Agricultural Sciences, Lanzhou, 730050 China; 8 Department of Applied Biotechnology, Molecular Biology Researches & Studies Institute, Assiut University, Assiut, 71526 Egypt; 9 Department of Animal Science, Center for Reproductive Biology and Health, College of Agricultural Sciences, Pennsylvania State University, University Park, PA 16802 USA

**Keywords:** Imprinted gene, *Gtl2*, PI3K/AKT/mTOR-ROS signaling, REDOX imbalance, Ancestral-like wool, Primary hair follicle

## Abstract

**Background:**

It is not uncommon for some individuals to retain certain primitive characteristics even after domestication or long-term intensive selection. Wild ancestors or original varieties of animals typically possess strong adaptability to environmental preservation, a trait that is often lacking in highly artificially selected populations. In the case of the Merino population, a world-renowned fine wool sheep breed, a phenotype with primitive coarse wool characteristic has re-emerged. It is currently unclear whether this characteristic is detrimental to the production of fine wool or whether it is linked to the adaptability of sheep. The underlying genetic/epigenetic mechanisms behind this trait are also poorly understood.

**Results:**

This study identified lambs with an ancestral-like coarse (ALC) wool type that emerged during the purebred breeding of Merino fine wool sheep. The presence of this primitive sheep characteristic resulted in better environmental adaptability in lambs, as well as improved fine wool yield in adulthood. Reciprocal cross experiments revealed that the ALC phenotype exhibited maternal genetic characteristics. Transcriptomic SNP analysis indicated that the ALC phenotype was localized to the imprinted *Gtl2*-miRNAs locus, and a significant correlation was found between the ALC wool type and a newly identified short Interstitial Telomeric Sequences (s-ITSs) at this locus. We further confirmed that a novel 38-nt small RNA transcribed from these s-ITSs, in combination with the previously reported 22-nt small RNAs cluster from the *Gtl2*-miRNAs locus, synergistically inhibited PI3K/AKT/Metabolic/Oxidative stress and subsequent apoptotic pathways in wool follicle stem cells, resulting in the ALC wool type. The necessity of *Gtl2*-miRNAs in controlling primary hair follicle morphogenesis, as well as the wool follicle type for ALC wool lambs, was verified using intergenic differentially methylated region-knockout mice.

**Conclusion:**

The ALC wool type of Merino sheep, which does not reduce wool quality but increases yield and adaptability, is regulated by epigenetic mechanisms in the imprinted *Gtl2*-miRNAs region on sheep chromosome 18, with the maternally expressed imprinted gene responsible for the ALC phenotype. This study highlights the significance of epigenetic regulation during embryonic and juvenile stages and emphasizes the advantages of early adaptation breeding for maternal parents in enhancing the overall performance of their offspring.

**Supplementary Information:**

The online version contains supplementary material available at 10.1186/s13578-023-01142-z.

## Background

Artificial selection of domesticated animals has increasingly emphasized the extreme pursuit of major economic traits, such as yield, which has led to the ultimate improvement of production efficiency, but also given rise to a prevalent issue—the conflict between increased yield and reduced environmental adaptability [1]. Despite efforts to discover more appropriate genotypes and aggregate favorable genes [2], significant progress in resolving this contradiction has yet to be achieved. Several recent studies have discussed the ab initio domestication of plants or animals with the idea of exploiting stronger adaptive traits from the ancestral varieties, and at the same time, directly achieve high-yield performance through hybridization, genome selection or gene editing [3]. It is true that ancestral species often possess advantageous adaptive characteristics. However, the genetic variants responsible for adaptation are frequently poorly understood, and adaptive traits can have a negative correlation with high-yielding genes [4, 5]. Balancing high yield with strong adaptability has emerged as a critical challenge in modern breeding practices.

According to Mendelian genetics, both male and female reproductive parents contribute equally to the genetic makeup of their offspring [6]. The common practice in breeding is to select equally excellent male and female livestock for breeding to improve the production performance of purebred progeny [7]. However, the inheritance of certain traits does not adhere to Mendelian laws, and the roles of the male and female parents may not be identical. Notable examples include the *Callipyge* muscle hypertrophy phenotype in sheep [7], which is determined by the imprinted *Dlk1*-*Gtl2* region on sheep chromosome 18 [8], and the *Callipyge* trait is more frequently inherited from the male parent [9]. Another example is seen in mammalian cloned embryos, where normal expression of the imprinted *Rtl1* from the male parent is necessary for successful early embryo development following transfer [10]. *Rtl1* is also an imprinted gene in the *Dlk1*–*Gtl2* region, which is equally important alongside the imprinted *IGF2*–*H19* region [11]. Variants in imprinted genes often cause very significant phenotypic changes [12, 13].

The breeding of fine wool sheep is a typical example of the contradiction between product improvement and environmental adaptation. Primitive domesticated sheep have two coats of fleece, with the outer coarse wool covering the shorter and finer inner wool. This type of wool is referred to as the “primitive wool characteristic” [14]. After thousands of years of domestication, people began to cultivate fine wool sheep varieties to obtain ultrafine wool fibers and obtain delicate and comfortable textiles [15]. After several hundreds years of breeding, we have obtained fine wool sheep represented by Merino sheep. But it has been found that when the wool fibers are too thin, the sheep will lose weight and the wool yield will decrease [16]. The lambs will be weaker and need special artificial care to improve their survival rate [17]. Research has shown that the primitive two coats of fleece exhibit better environmental adaptability, such as cold resistance and moisture resistance, compared to lambs with homogeneous fine wool throughout their body [18, 19]. Is there a sheep in reality that has both excellent wool quality and good environmental adaptability? In modern fine (MF) wool sheep populations, lambs with primitive wool characteristics can occasionally be found [20], which we refer to as the ancestral coarse wool (ALC) type. These ALC wool lambs exhibit better growth performance and environmental adaptability than homogeneous fine wool lambs [18]. However, the potential relationship between wool type and environmental adaptability is still unknown, and its genetic mechanism needs to be explored.

In this study, we found that ALC lambs exhibited superior adaptation, displaying increased body weights, enhanced wool yields and better wool quality as they matured compared to MF lambs. The ALC wool type is regulated by epigenetic mechanisms in the *Gtl2*-miRNAs imprinted gene cluster, wherein the maternally expressed imprinted gene assumes responsibility for the ALC phenotype. These findings provide a groundbreaking perspective on the utilization of epigenetic mechanisms in modern breeding strategies, opening up new possibilities for enhancing desired traits and improving overall breeding outcomes.

## Results

### The discovery of ALC wool lambs with better production performance as adults

**Fig. 1:**
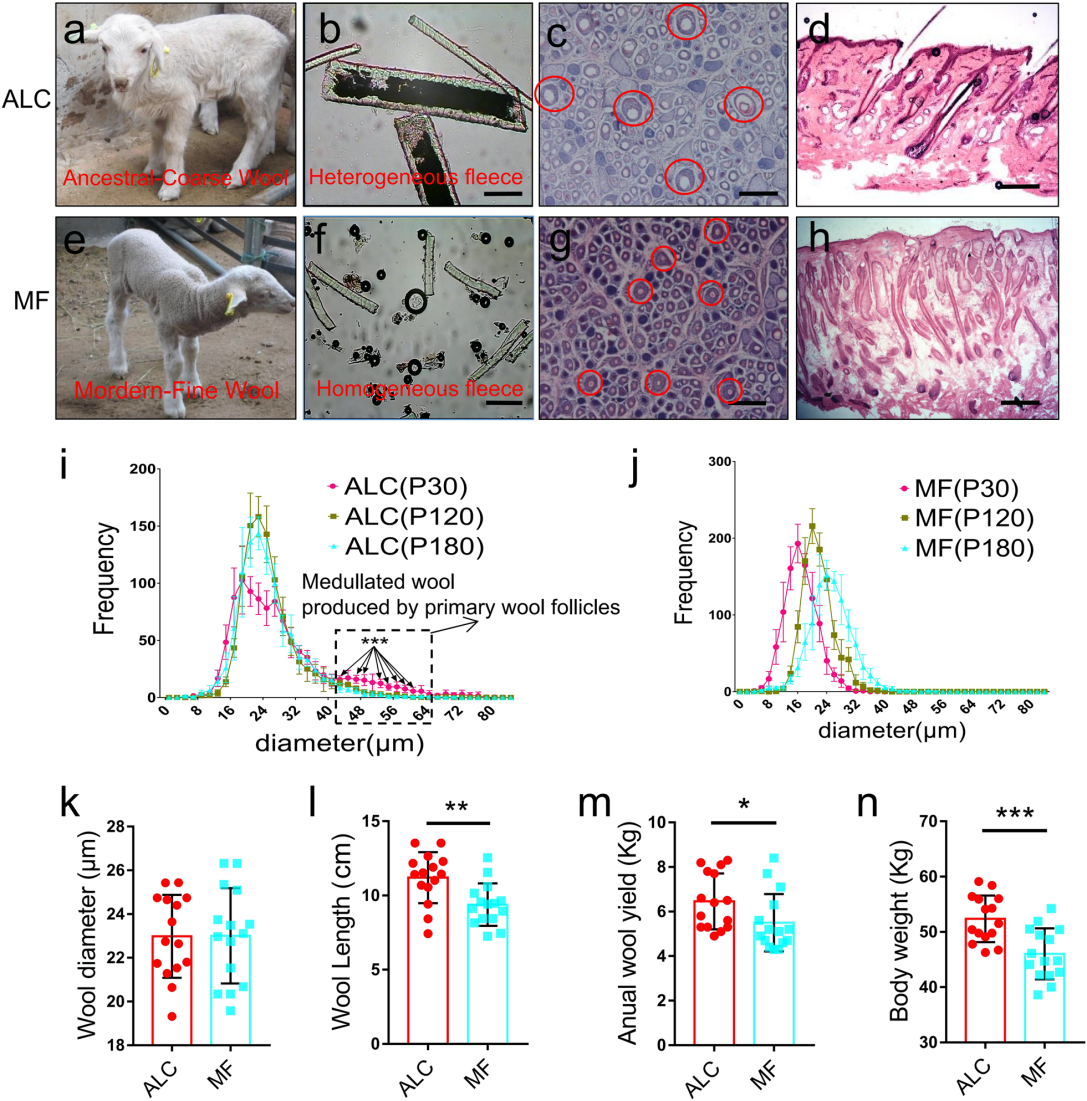
Discovery of ancestral-like coarse (ALC) wool characteristics in newborn Merino sheep. **a**–**d** The phenotypic properties of newborn ALC wool sheep (P30) The red circles in **c** indicates primary wool follicles of ALC lambs. **e**–**h** The phenotypic properties of MF wool newborn sheep (at P30). The red circles in **g** indicates primary wool follicles of modern fine (MF) wool lambs. **i**, The distribution of ALC wool diameters (line graph) at three different stages (P30 [30 d postpartum], P120, and P180). **j** The distribution of MF wool diameters (line graph) at three different stages (P30, P120, and P180). The bar is 22 μm, The bar in **D** and **E** are 55 μm, The bar in **E**, **F**, **I** and **J** are 100 μm. **k** The wool diameter of ALC and MF wool sheep in adulthood (1 year old, n = 15). **l** The wool length of ALC and MF wool sheep in adulthood (1 year old, n = 15, **P < 0.01). **m** The annual wool yield of ALC and MF wool sheep in adulthood (1 year old, n = 15, *0.01 < *P* < 0.05). **n** The body weight of ALC and MF wool sheep in adulthood (1 year old, n = 15, ***P < 0.001)

We have identified lambs with the ALC wool type in a Chinese Merino fine wool flock in Multiple Ovulation and Embryo Transfer (MOET). Compared to MF wool lambs covered in fine curly fleece, ALC wool lambs have straight and coarse wool (Fig. 1a, e). Follow-up observations showed that the ALC wool type only occurred at birth, and a large number of primary wool follicles with coarse wool bulbs are present in the skin tissue of ALC wool lambs (Fig. 1c, d), and grow coarse wool filled with medulla in the wool shaft (Fig. 1b). However, over time, this distinctive ALC wool type gradually diminishes between 30 and 120 days after birth, and by 180 days the medullated coarse wool disappears completely (Additional file 1: Fig. S1a, c). Interestingly, in the wool diameter distribution graph, the main peak of ALC wool diameter did not change significantly over the three periods (Fig. 1i). In the MF lambs, the coat always consisted of fine wool (Fig. 1e–h, Additional file 1: Fig. S1b, d) and the wool diameter increased gradually with age (Fig. 1j). Comparing the wool diameters of the ALC and MF groups at 12 months of age, there is no longer a significant difference (Fig. 1k), suggesting that the ALC wool type at a young age does not reduce the wool quality at adulthood. In addition, ALC wool lambs weighed more than MF at birth and maintained this advantage into adulthood (Fig. 1n, Additional file 1: Fig. S7d), and at this time, ALC sheep had significantly greater wool length and grease wool yield than MF sheep (Fig. 1l, m). Thus, ALC lambs exhibit a coarse wool type at birth and transform into fine wool type with better production characteristics than adult MF sheep.

### The ALC wool type exhibits exclusive maternal inheritance of major genes at the imprinted *Gtl2*-miRNA locus

**Fig. 2:**
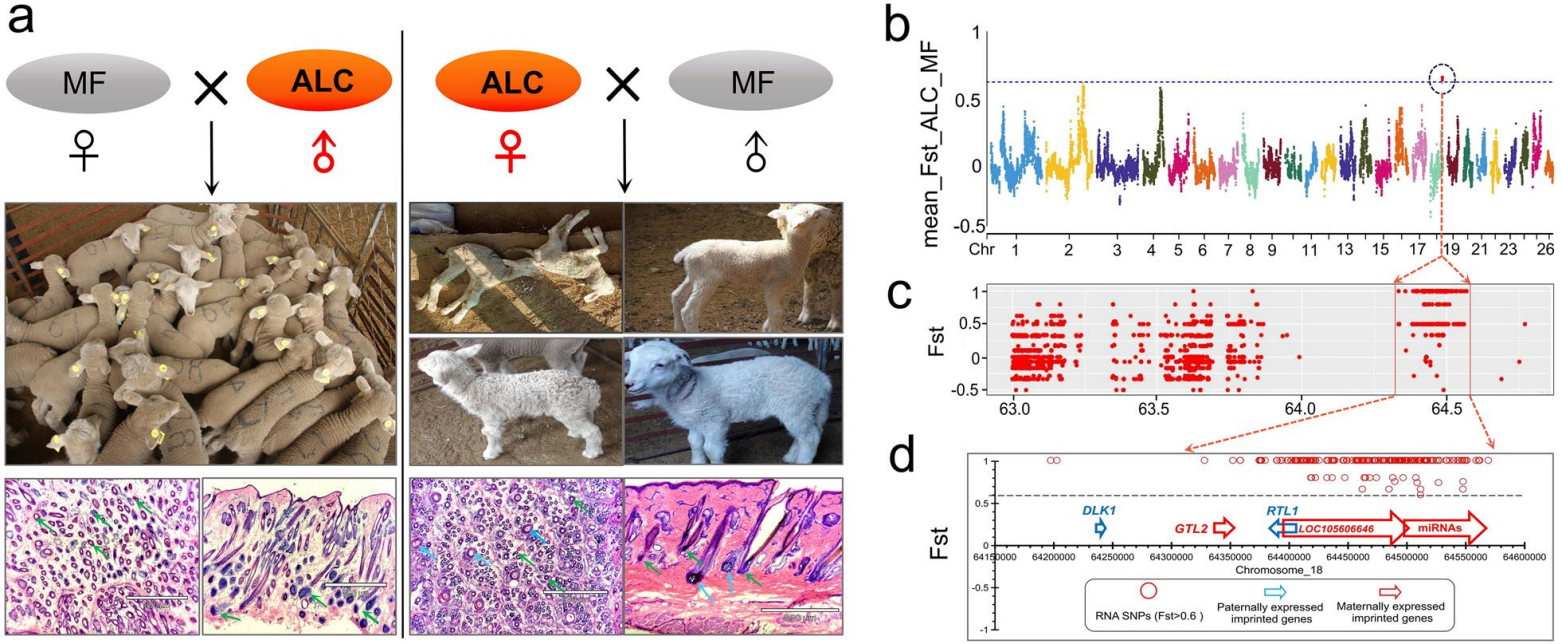
ALC wool type demonstrated strictly maternal inheritance of major genes at the *Gtl2*-miRNA locus. **a** Reciprocal cross hybrids between ALC and normal Merino sheep. The blue arrow indicates the primary hair follicles. The green arrow indicates secondary follicles. The scale bar is 890 μm. **b** The transcriptomic selection signals between ALC and MF wool lambs (Chr18: 63900001–64500000 [GCA_000298735.2 Oar_V4.0]) (Fst > 0.6). **c** Signals of candidate region calculated by FST. **d** The candidate region for ALC wool in Merino lambs located in the imprinted gene cluster (*Dlk1*-*Gtl2* locus)

As the ALC wool type occurs only at the lamb stage, we selected skin tissues from same-sex half-sibling ALC (n = 3) and MF wool lambs (n = 3) for transcriptome sequencing (Additional file 2: Table S1). Fst analysis of transcriptomic SNPs data showed that the strongest selection signal was concentrated on chromosome 18: 63900001–64500000 (GCA_000298735.2, Oar_V4.0) (Fst > 0.6) (Fig. 2b, c). Notably, this candidate locus is located in an imprinted gene cluster containing paternally expressed imprinted genes (*Dlk1*, *Rtl1* and *Dio3*) and maternally expressed multiple non-coding RNAs (ncRNAs) such as the long non-coding RNA (lncRNA) *Gtl2*, *Loc105606646* and miRNA clusters (Fig. 2d). Further analysis showed that the distribution of selection signals in the imprinted regions of *Dlk1*-miRNAs favored the maternally expressed imprinted gene cluster *Gtl2*-miRNAs (Fig. 2d).

To verify whether the ALC trait fits the imprinted genetic model, we used adult ALC and MF sheep in a reciprocal crosses test to explore the genetic pattern of the ALC wool trait, and the result showed that when ALC sheep were crossed as the female parent, all five lambs showed heterogeneous wool consistent with the female parent, i.e. the ALC wool type (Fig. 2a, right). In contrast, when the ALC wool sheep were used as the male parent, all 42 lambs produced had homogeneous fine wool, consistent with the MF wool type (Fig. 2a, left). The results of the reciprocal crosses test support the imprinted genetic pattern that the ALC wool type is of maternal origin. Fst analysis of transcriptomic SNPs data (n = 5 vs. 5) showed that the strongest selection signal was also concentrated on chromosome 18: 63900001–64500000 (GCF_016772045.2, ARS-UI_Ramb_v2.0) (Fst > 0.6) (Additional file 1: Fig. S9). This leads to the inference that the maternally expressed imprinted gene cluster, *Gtl2*-miRNAs, is a candidate locus for the ALC lamb wool type.

### Maternally expressed imprinted genes in *Gtl2*-miRNAs locus are correlated with the ALC phenotype

**Fig. 3:**
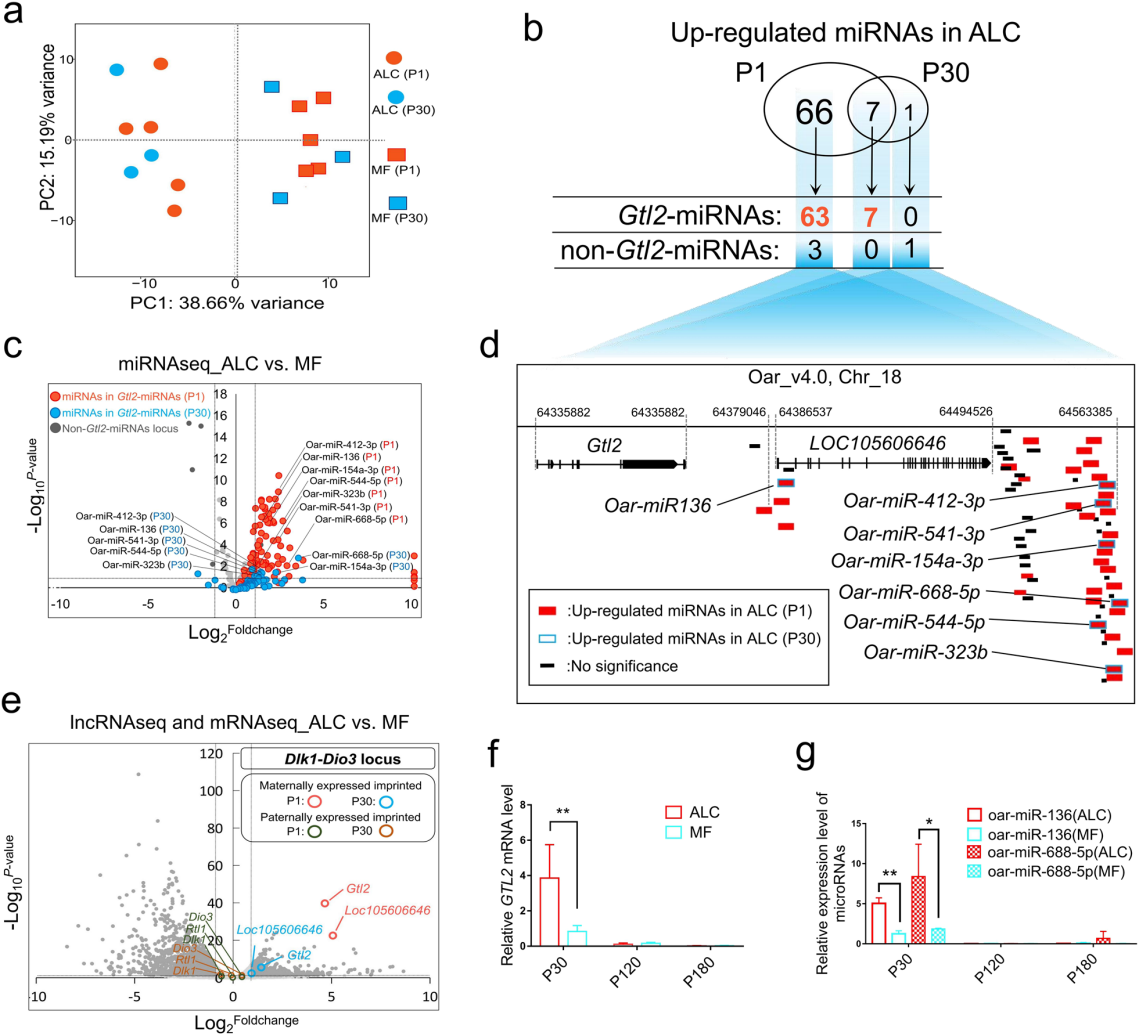
Comprehensive comparison and global analysis of differentially expressed ncRNAs between P1 and P30 stages. **a** Principal Component Analysis of miRNA data in ALC and MF lambs at P1 and P30. **b** Venn diagram of significantly upregulated miRNAs in ALC at P1 and P30. **c** Volcano plots of DE miRNAs between ALC and MF lambs at P1 and P30. **d** Distribution of all significantly upregulated miRNAs in ALC lambs at the *Gtl2*-miRNAs locus. **e** Volcano plots of DE mRNA and lncRNA between ALC and MF lambs at P1 and P30. **f** The expression level of *Gtl2* in different growth stages measured by qPCR (n = 3, **P < 0.01). **g** The expression level of two candidate miRNAs in P30, P120, and P180 detected by qPCR (n = 3, *P < 0.05, **P < 0.01)

The ALC phenotype was evident at birth and the proportion of coarse wool at 30 days postnatal (P30) was significantly reduced but the phenotype remains, however, the proportion of coarse wool fibers is significantly lower. Therefore, we sequenced mRNA, miRNA and lncRNA from the skin tissue of ALC lambs at postnatal day 1 (P1) and P30 and their control MF lambs (Additional file 2: Table S1). All paternally expressed imprinted genes in this region did not show significant differences between the ALC and MF wool type groups (Fig. 3e). Differential expression was concentrated in the cluster of maternally expressed non-coding RNA (*Gtl2*-miRNAs), with significant differences in the expression of lncRNA *Gtl2* and *LOC105606646* (Fig. 3e), and the cluster of miRNAs in this region was more significantly differentially expressed (Fig. 3c). Specifically, miRNA principal component analysis (PCA) showed that ALC and MF wool type lambs at both P1 and P30 were clearly distinguished in the first principal component (Fig. 3a). A total of 73 miRNAs were up-regulated in the skin of ALC wool lambs on day 1 after birth, 70 of which were located in the *Gtl2*-miRNA region, accounting for 65% of all maternally expressed imprinted miRNAs in this imprinted region (Fig. 3b, d). Eight miRNAs were differentially expressed in the skin tissue of ALC and MF wool lambs at P30, seven of which were up-regulated and all the seven up-regulated miRNAs were located in the *Gtl2*-miRNAs region (Fig. 3b–d). In addition, as the phenotypic difference between ALC and MF decreased during P30 compared to P1, the differential expression multiplier of miRNAs also decreased, as did the significance of the difference (Fig. 3c). The expression of the *Gtl2* gene and miRNAs clusters in the skin tissue of ALC sheep also decreased to very low levels, a trend consistent with the phenotypic changes (Fig. 3f, g). These results again demonstrate that the maternally expressed *Gtl2*-miRNAs gene cluster is highly correlated with the ALC phenotype and should be considered as a major candidate gene.

### A newly identified short interstitial telomeric sequences in *Gtl2*-miRNA locus (*Gtl2*-sITSs) is a potential regulatory element regulating the production of ALC wool lambs

**Fig. 4:**
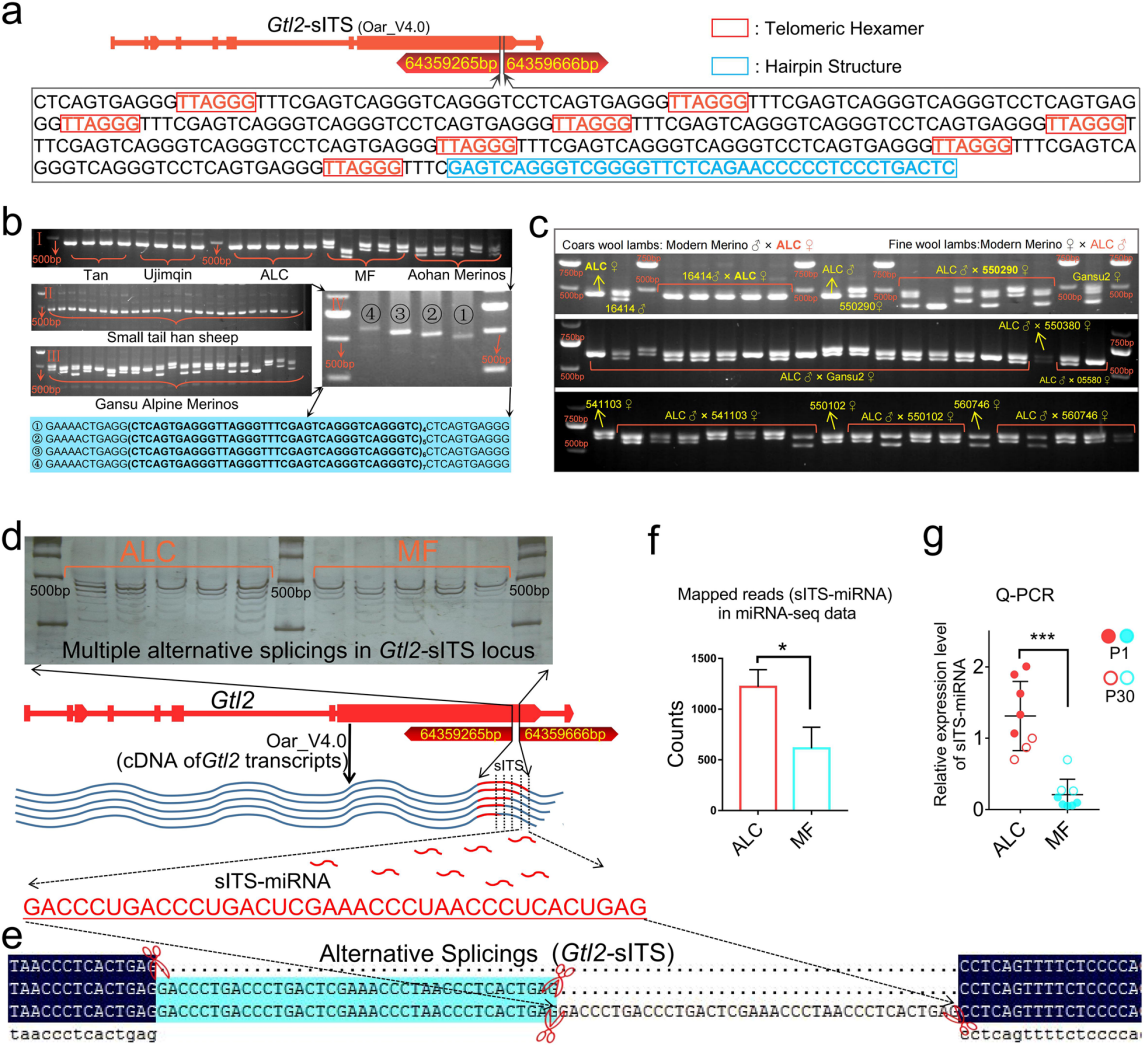
A newly identified s-ITS in exon of *Gtl2*. **a** The repetitive sequence of the *Gtl2* gene showed typical features of short interstitial telomeric sequences (sITSs). The short sequence in red font is telomere DNA, and the sequence in blue font is the hairpin structure. **b** Genotyping of the repeat element (RE) mutation in DNA extracted from skin tissue of different sheep breeds. (I) Genotypes of the RE mutations of Tan sheep (ALC wool), Ujimqin sheep (ALC wool), ALC (ALC wool), MF (MF wool), and Merinos (MF wool). (II) RE genotypes of Small Tail Han sheep (ALC wool). (III) RE genotypes in Merino sheep (MF wool). (IV) Allele types of REs identified using sub-PCR. The four alleles were sequenced as shown below. M: Marker. Repeat sequence units are represented in a large, bold font. The number of repetitions of the base unit is shown as the numbers in the lower right of the repeating unit (below). Primers are listed in Additional file 2: Table S5, named “CNV identification_1.” ① to ④ indicate the different copy number of s-ITS in one allele, specifically, it represents the number of copies from 4 to 7. **c** CNV analysis of sITSs in reciprocal cross families. Primers are listed in Additional file 2: Table S5, named “CNV identification_2.” The yellow lettering in the diagram represents ear tag of sheep, where ALC indicates the ancestral-like coarse wool sheep. **d** Multiple alternative splicing of the *Gtl2* gene was detected using polyacrylamide gel electrophoresis; the primers are shown in Additional file 2: Table S5 and named “CNV identification_1.”, the yellow lettering in the diagram represents ear tag of sheep, where ALC indicates the ancestral-like coarse wool sheep.** e** The generation process pattern of *sITS*-miRNA in the *Gtl2*-miRNA locus. G, DNAMAN multiple sequence alignment analysis was used to analyze the result of Sanger Sequencing for three adjacent alternative splicing of the *Gtl2*-sITS site. **f** The comparative analysis of *sITS*-miRNA expression using miRNA-seq data (*, 0.01 < *P* < 0.05). **g** Comparative analysis of *sITS*-miRNA expression in ALC and MF wool lambskin tissue at P1 (n = 5) and P30 (n = 3). (***P < 0.001)

Sequence alignment of *Gtl2*-miRNA region revealed that the most obvious difference between ALC and MF at the genomic level occurs in a unique repeat element (RE) in the exons of the *Gtl2* gene that contains a telomeric hexamer (TTAGGG) in each repeat unit, a type of RE known as short interstitial telomeric sequences (s-ITSs) (Fig. 4a) [21, 22]. The s-ITSs within the *Gtl2* gene are the first s-ITS sequences identified in sheep, and we have uploaded them to the GenBank database under accession number ON512716. *Gtl2*-sITSs show genomic variation between ALC and MF wool type lambs, with homozygote corresponding to the ALC wool type and heterozygotes to the MF wool type (Fig. 4b). In addition to the ALC and MF wool type lambs, we also examined the genotypes of *Gtl2*-sITSs in several coarse and fine wool breeds (n = 314), with 96.22% homozygote in the coarse wool breeds and 92.93% heterozygotes in the fine wool breeds (Table 1, Additional file 1: Fig. S2b). *Gtl2*-sITSs corresponded to a copy number of 12 in both ALC and coarse wool breeds, while the copy numbers ranged from 8 to 14 in MF and fine wool breeds (Table 1). In particular, in the aforementioned reciprocal crosses test, when the ALC wool type sheep with homozygote was the female parent and the normal Merino ram with heterozygotes was the male parent, all five lambs from the crosses formed homozygote with the same band size as their female parent (Fig. 4c). Conversely, when the ALC wool type sheep (homozygote) was used as the male parent and the heterozygous normal merino ewe was used as the female parent, 37 of the 42 offspring lambs obtained were heterozygotes and 5 were homozygote, and the homozygote did not match their male parent (Fig. 4c). Clearly, the genotypes of the *Gtl2*-sITSs corresponded stably to the ALC phenotype and were consistent with the transmission pattern of maternal imprinted inheritance (Fig. 4c).

**Table 1:** The genotypes of the Gtl2-sITSs locus showed a distinct difference between coarse and fine wool sheep breeds

Breed	Genotyped animals (n)	Wool phenotype	Genotype			
			Heterozygote		Homozygote	
			Copy number	Proportion	Copy number	Proportion (%)
Tan	39	Coarse	13	5.13%	12	94.87
STH	30	Coarse	0	0	12	100
Ujimqin	37	Coarse	13	5.41%	12	94.59
ALC	5	Coarse	0	0	12	100
MF	5	Fine	9 to 13	100	0	0
AuM	60	Fine	8 to 14	95%	8 to 14	5
AoM	50	Fine	8 to 14	88%	8 to 14	12
GaM	88	Fine	8 to 14	94.32%	8 to 14	5.68

*Tan* Tan sheep, *STH* Small Tail Han sheep, *Ujimqin* Ujimqin sheep, *AuM* Australian Merino, *AoM* Aohan Merino, *GaM* Gansu alpine Merino

Multiple novel transcripts of the *Gtl2*-sITSs locus with sequentially decreasing sequence length were identified by reverse transcription polymerase chain reaction (RT-PCR) and analyzed by polyacrylamide gel electrophoresis and silver staining (Fig. 4d). Using PCR, cloning and sequencing, all of these transcripts were found to differ from each other by 38 bases or 38-base multiples (Fig. 4e). We referred to this 38-base RNA repeat unit as sITS-miRNA. Reads data for sITS-miRNA were also obtained in the miRNA-seq data and, in the ALC wool lambs, sITS-miRNA expression levels were significantly higher than in the MF lambs (Fig. 4f), a result confirmed by Q-PCR (Fig. 4g).

Notably, a comparative search tool (BLAST) used at NCBI to analyze the sequence similarity of *Gtl2*-sITSs between species (Additional file 1: Fig. S2a) revealed that *Gtl2*-sITSs were only present in ruminants and marine mammals, and further analysis revealed that the earlier homologous sequence of this sITSs was from bony fish (Additional file 1: Fig. S2a, c). This suggests that *Gtl2*-sITSs may be an ancient locus preserved from the ruminant ancestor and may bring about a unique regulatory pattern in ruminants.

### *Gtl2*-miRNAs’ role in primary hair follicle development was confirmed by intergenic differentially methylated region (IG-DMR) knockout (KO) within the *Dlk1-Meg3* locus in mice

**Fig. 5:**
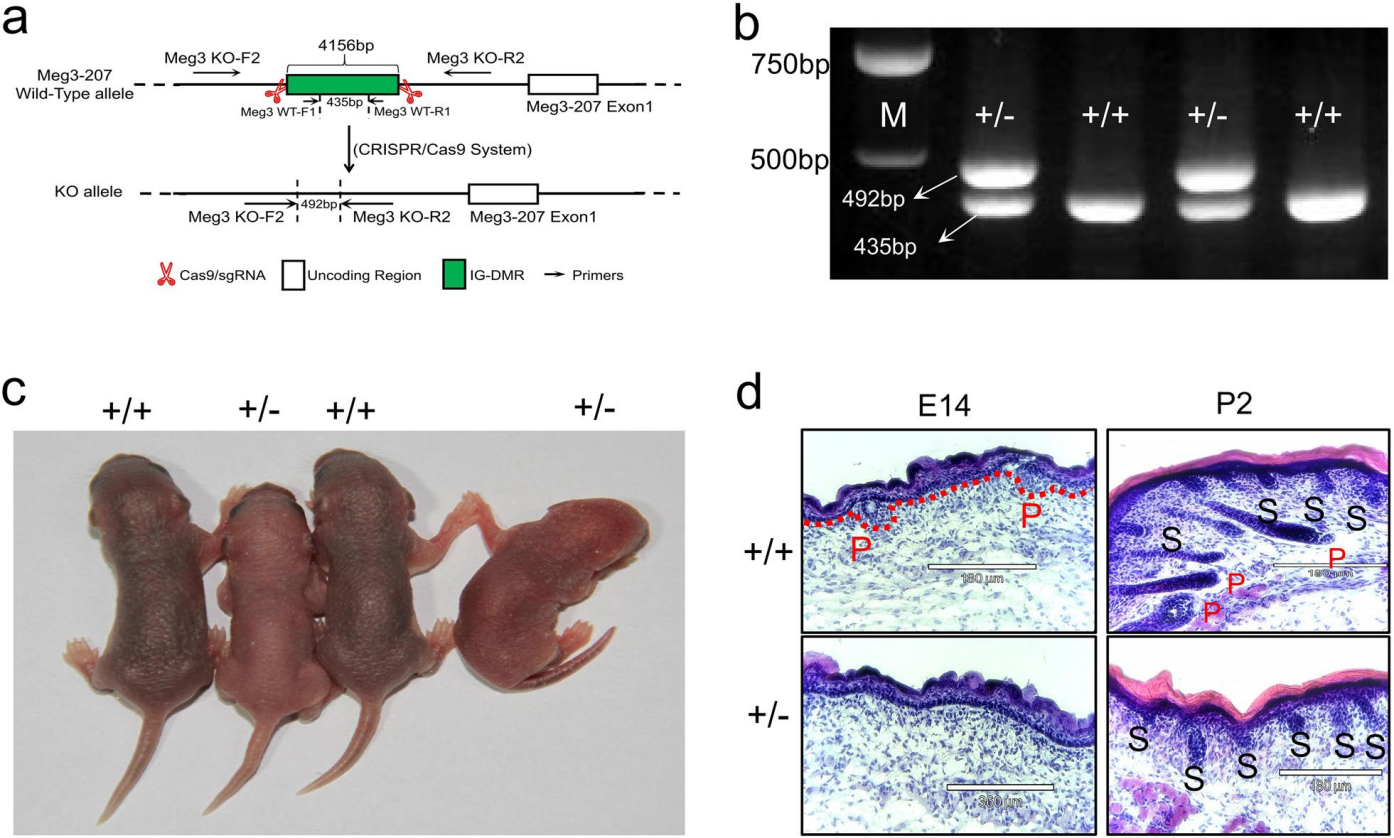
*Meg3* (IG-DMR)-knockout (KO) mice showed an essential role of *Gtl2* (*Meg3*)-miRNAs in controlling primary hair follicle morphogenesis. **a** Preparation and identification strategy of *Meg3* (IG-DMR)-KO mice. **b** Identification of *Meg3* (IG-DMR)-KO mice by polymerase chain reaction along with the primers (shown in Additional file 2: Table S5). **c** Appearance of *Meg3*(IG-DMR)-KO mice at P2. **d** Observations on the morphologic structure of hair follicles in skin tissues of KO and NC mice

The miRNA sequence of the *Gtl2*-miRNAs locus is highly conserved and the locus exhibits maternal expression pattern in mammals [23]. The *Gtl2* locus is known as *Meg3* in mice [24]. The previous results showed that high expression level of ncRNAs in *Gtl2*-miRNAs loci was highly associated with the ALC wool trait, which is generated by primary wool/hair follicles. To validate this result, we constructed KO mice with *Meg3*(*Gtl2*)-IG-DMR knocked out on the maternal chromosome to silence ncRNA clusters originating from the maternal line (Figs. 5a, 8a–c), based on previous studies [25–27], heterozygous offspring carrying maternal DMR-KO were generated on embryonic day 14 (E14) and postnatal day 2 (P2) (Figs. 5b, c, 8a, Additional file 2: Table S6). E14 is a critical time point for primary hair follicle morphogenesis [28], and skin histological analysis showed that wild-type mice at E14 had formed hair germs and hair buds, whereas sibling KO mice of the same sex did not have these features (Fig. 5d). The skin color of P2 stage KO mice was lighter than that of wild-type fetuses of the same sex (Fig. 5c). Skin color reflects the different hair follicle development status [29]. wild-type mice (P2) have formed primary hair follicles, KO mice have no primary hair follicles in their skin, but their secondary hair follicles develop normally (Fig. 5d). *Meg3*(*Gtl2*)-miRNA expression is silenced in KO mice (Fig. 8c), and their hair follicle development pattern is consistent with that of MF sheep at birth. The hair follicle development pattern in wild-type mice with normal *Meg3*(*Gtl2*)-miRNA expression (Fig. 8c) was consistent with that of birth-period ALC sheep. The necessity of *Gtl2*-miRNAs for primary hair follicle development was confirmed.

### sITS-miRNA and a cluster of miRNAs within the *Gtl2*-miRNA locus, collaboratively inhibit the PI3K/AKT/mTOR/ROS pathway in ALC lamb skin tissue

**Fig. 6:**
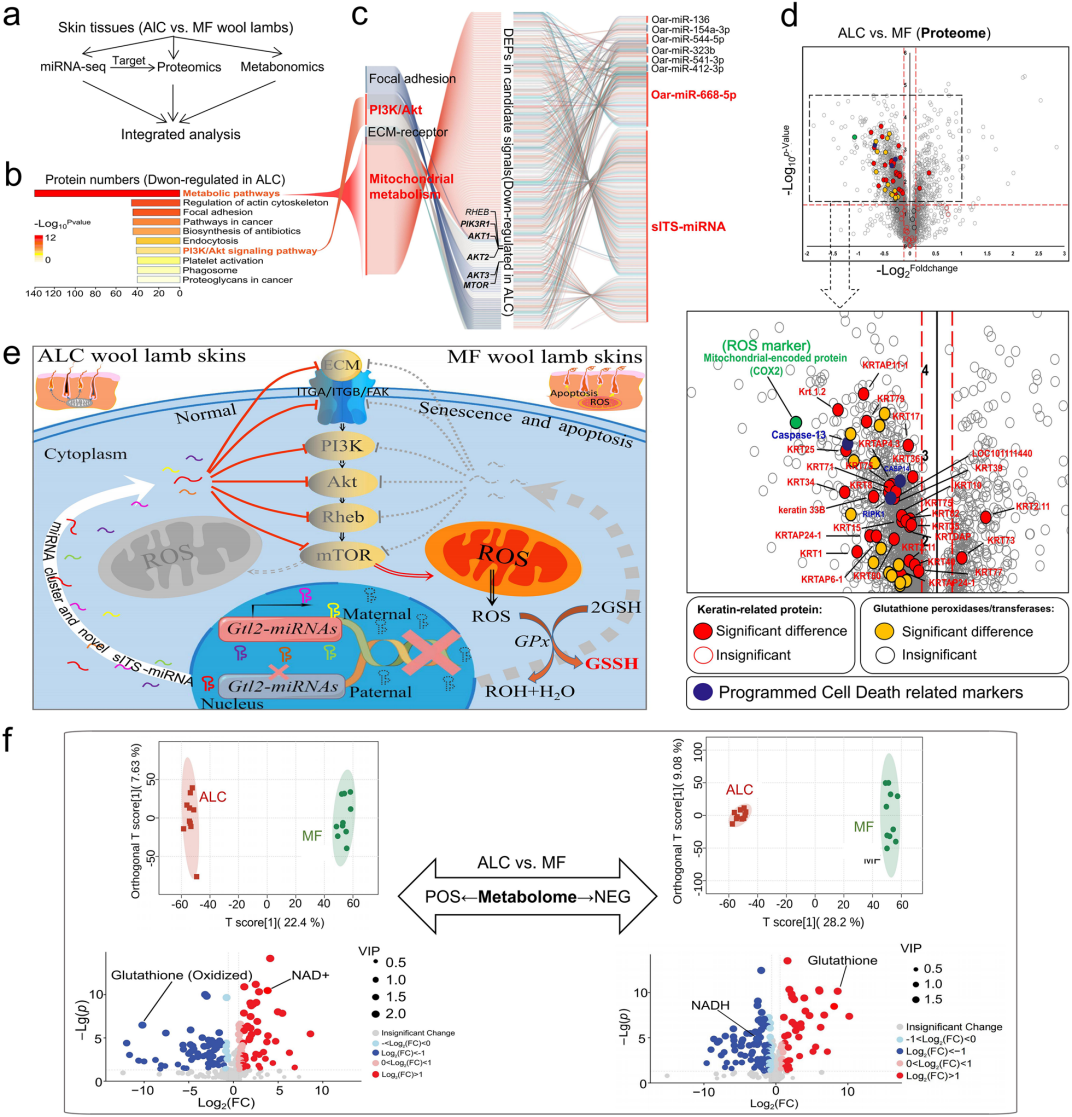
Multiple omics analysis revealed the candidate signaling pathway (PI3K/AKT/mTOR/ROS). **a** The flow chart for multiple omics integrative analysis of miRNA-seq, the proteome, and the metabolome. **b** Functional classification of downregulated proteins in ALC wool lambskin at P30. **c** The differentially expressed miRNAs and their potential capacity to target candidate pathways. **d** Volcano plot of differentially expressed proteins between ALC and MF wool lamb at P30. **e** Model depicting the role of *Gtl2*-miRNA locus in inhibiting the PI3K-AKT-mTOR pathway and the subsequent mitochondrial metabolism. The model was drawn using Figuredraw (www.Figuredraw.com). **f** OPLS-DA scores scatter plot of ALC and MF groups in the positive and negative ion mode (POS and NEG)

**Fig. 7:**
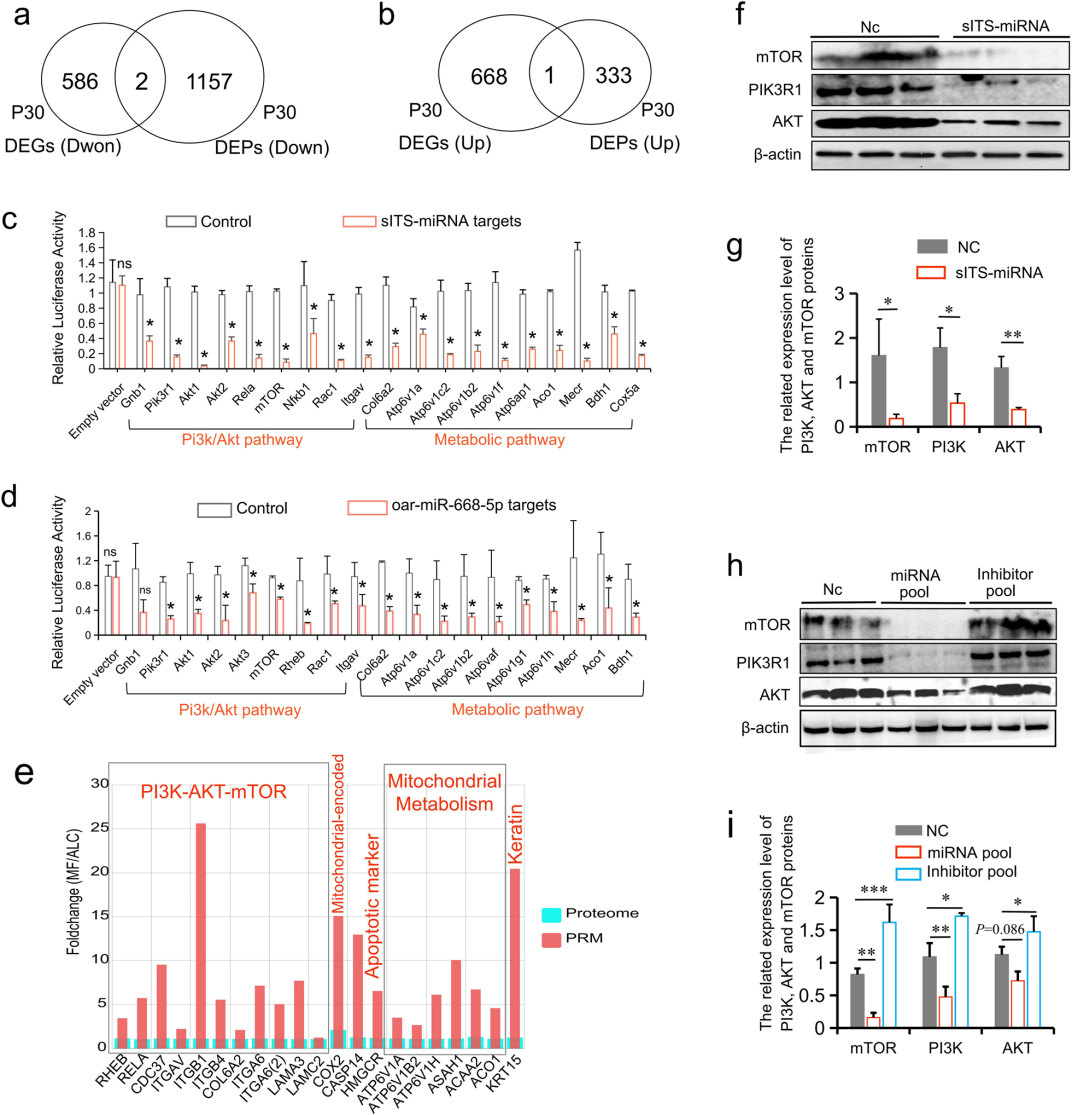
Further experiments carried out to verify the candidate signaling pathway. **a** The overlap of upregulated DEGs and differentially expressed proteins (DEPs) in ALC wool skin tissue at P30. **b** The overlap of downregulated DEGs and DEPs in ALC wool skin lamb tissue at P30. **c** The luciferase reporter assays were performed to determine functional *sITS*-miRNA target sites. **d** The luciferase reporter assays were performed to determine functional *oar-miR-668-5p* target sites. **e** The PRM-MS results of selected DEPs in the validation cohort (n = 3, *P* < 0.05). **f** Western blot analysis of the PI3K/AKT/mTOR signaling pathway with overexpressed *sITS*-miRNA and its negative control in sheep fetal fibroblast cells. **g** Relative expression level of mTOR, AKT, and PI3K protein after overexpressing *sITS*-miRNA and its negative control in sheep fetal fibroblast cells (grayscale analysis) using ImageJ software, *P < 0.05, **P < 0.01. **h** Western blot analysis of PI3K/AKT/mTOR signal pathway with the overexpressed miRNA pool, inhibitor pool, and negative control in sheep fetal fibroblast cells. **i** Relative expression level of mTOR, AKT, and PI3K protein after overexpressing the miRNA pool, inhibitor pool, and negative control in sheep fetal fibroblast cells (grayscale analysis) using ImageJ software, *P < 0.05, **P < 0.01, ***P < 0.001

To investigate the molecular mechanisms of miRNAs in the *Gtl2*-miRNA region, skin samples from ALC and MF wool lambs were divided into three parts—one for RNA-seq, one for the proteome and subsequent validation experiments (parallel reaction monitoring, PRM), and the other one for the metabolome (Fig. 6a). The results showed that the differentially expressed genes (DEGs) and differentially expressed proteins (DEPs) between the two groups overlapped by only two down-regulated genes and one up-regulated gene (Fig. 7a, b), implying that the regulation of many genes occurs post-transcriptionally. Prediction and functional enrichment analysis of miRNA target genes showed that the main signaling pathways regulated by up-regulated miRNAs in the *Gtl2*-miRNAs region were PI3K-AKT and metabolic signaling pathways (Additional file 1: Fig. S6a–c). Furthermore, based on TMT-Proteome, we found that down- regulated DEPs in ALC wool lamb skin were also mainly enriched in the Metabolic and PI3K-AKT signaling pathways (Fig. 6b, c and Additional file 1: Fig. S8). PRM analysis also confirmed the down-regulated expression of this pathway marker gene (Fig. 7e). This suggests that translation of genes related to the Metabolic and PI3K-AKT signaling pathways is suppressed in ALC wool lamb skin tissue (Fig. 6e).

miRNA target gene prediction (miRanda) indicates that the main signaling pathways regulated by miRNAs in the *Gtl2*-miRNAs region are PI3K-AKT and metabolic signaling pathways (Additional file 1: Fig. S6a–c), and the seven miRNAs with high expression in the ALC group that overlapped in the P1 and P30 phases were all able to target and regulate these PI3K-AKT and Metabolic signaling pathways, the one with the highest targeting ability and the highest number of target genes was *oar-miR-668-5p* (Fig. 6b, c). The newly identified *sITS-*miRNA in this study had most target genes (Fig. 6c, Additional file 2: Tables S2, S3). The luciferase reporter assay verified the targeting relationship between *sITS-*miRNA and *oar-miR-668-5p* and the above pathway genes (Fig. 7c, d). In sheep fetal fibroblasts transfected with a library of miRNAs (seven P1 and P30 overlapping DE miRNAs) and *sITS-*miRNA, the markers (mTOR, PI3K, and AKT proteins) in PI3K-AKT-mTOR pathway were significantly down-regulated (Fig. 7f–i), demonstrating that miRNAs at the *Gtl2*-miRNA loci play an important role in the translational regulation of PI3K-AKT-mTOR pathway-related genes.

The results of fluorescence in situ hybridization (FISH) showed that *Gtl2*-miRNAs were highly expressed in the dermal papilla cells of primary wool follicles in ALC lambs (Fig. 9a, left), which contains abundant stem cell-like cells responsible for wool follicle regeneration and hair follicle cycle [30]. *Gtl2*-miRNAs were not expressed in the dermal papilla cells of the MF group (Fig. 9a, left). Furthermore, the PI3K-AKT-mTOR pathway markers AKT1, 2, 3, and mTOR were highly expressed in the dermal papilla cells of the MF group but not expressed in the dermal papilla cells of the ALC group (Fig. 9a, left). This shows a negative correlation between *Gtl2*-miRNA expression and PI3K-AKT-mTOR pathway activity. TUNEL (terminal deoxynucleotidyl transferase dUTP nick end labeling) staining was used to detect cell apoptosis, and more positive signals were detected in the skin of the MF group than that of the ALC group, especially in the dermal papilla cells (Fig. 9a, right). Additionally, when Ki67 staining was used to observe cell proliferation, the opposite trend was observed, with more proliferation signals in the primary wool follicles of ALC wool lambs skin than in MF wool lambs (Fig. 9b, left). Staining for the oxidative stress marker 8-OHDG also showed that dermal papilla cells of the MF wool lambs had abundant positive signals, while no oxidative stress signals were detected in dermal papilla cells of the ALC lambs (Fig. 9a, middle). Under transmission electron microscopy (TEM), the most prominent differences appeared in the dermal papilla cells (Fig. 9b, right). Specifically, the fuzzy cell contours and chromatin condensation in the cell nucleus in the dermal papilla cells of MF wool type lambs indicate that the cell has features of programmed cell death (Fig. 9b, right) [31]. These results indicate that the up-regulation of the PI3K-AKT-mTOR-ROS pathway in the dermal papilla cells of the MF group resulted in more obvious aging and apoptosis. Apoptotic signals occurred in the wool follicle stem cells of the MF group.

**Fig. 8:**
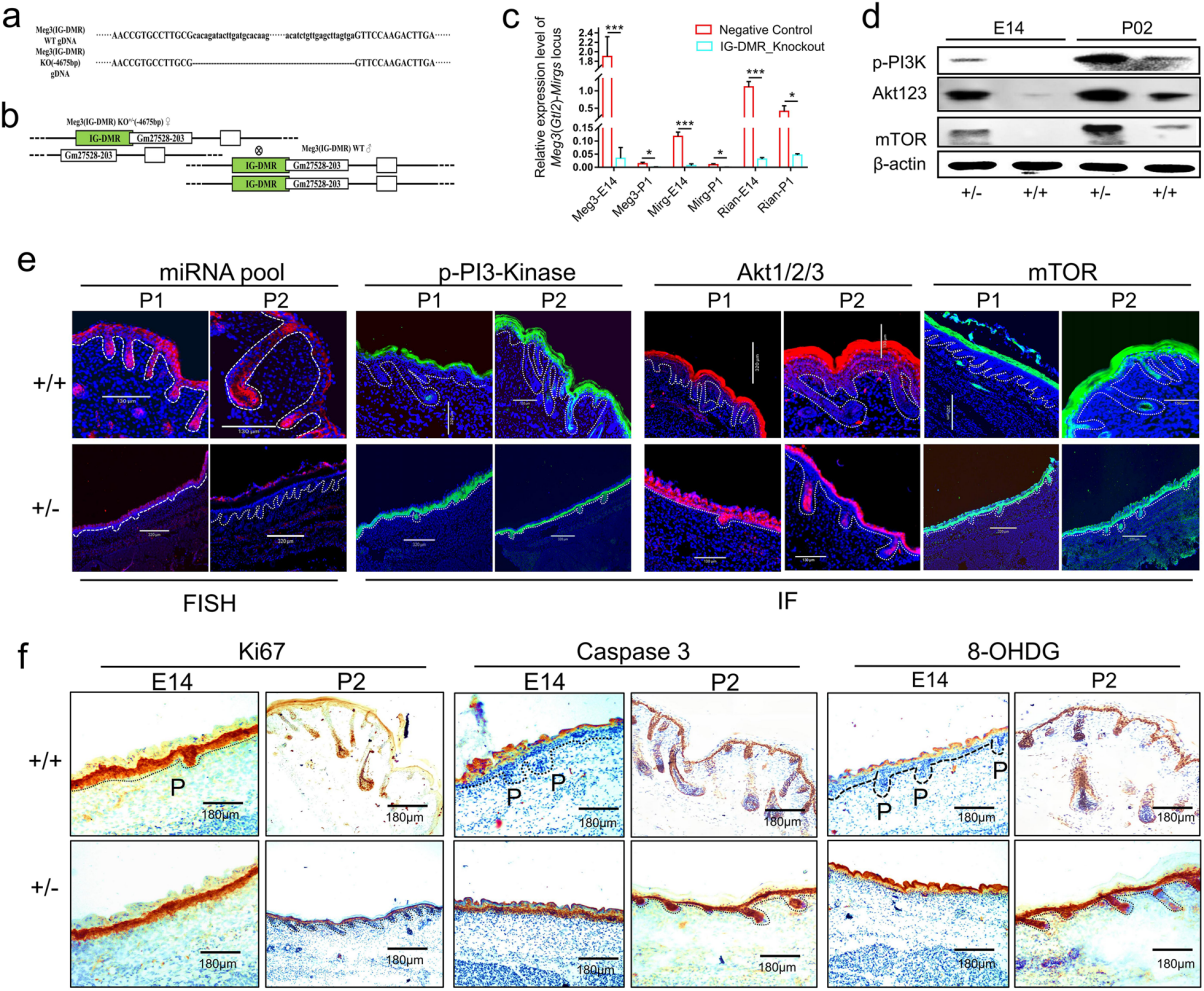
Further experiments carried out to verify the candidate signaling pathway using *Dlk1*-*Meg3* (IG-DMR)-knockout (KO) mice model. **a** Alignment of the genomic DNA sequence of *Meg3* (IG-DMR)-wild-type and -KO (− 4675 bp) mice. **b** Breeding scheme to generate *Meg3* (IG-DMR) maternally derived KO mouse heterozygotes by crossing KO heterozygous females with WT males. **c** The relative expression level of maternally expressed imprinted non-coding RNAs at the *Meg3*(*Gtl2*)-miRNAs locus. The primers are shown in Additional file 2: Table S5. **d** Western blot detection of the difference in PI3K/AKT/mTOR signaling pathway activity between KO and non-KO mice littermates with the same sexes at E14 and P2. **e** Activity of the PI3K/AKT signaling pathway in longitudinal sections of *Meg3* (IG-DMR)-KO mouse skin tissue as determined using immunohistochemical staining. The letter P indicates the primary hair follicle. **f** Detection of cell apoptosis, proliferation and oxidative stress levels in longitudinal sections of *Meg3* (IG-DMR)-KO mouse skin tissue by using Ki67, Caspase 3 and 8-OHDG immunohistochemical staining, respectively. The letter P indicates the primary hair follicle

The *Meg3* (*Gtl2*)-DMR-KO mouse verified the targeting relationship between ncRNAs and the PI3K-AKT-mTOR pathway. After the ncRNAs expressed in the maternal origin were silenced in the KO mice (Fig. 8c), the PI3K-AKT-mTOR pathway was significantly activated (Fig. 8d, e). E14 is the developmental stage of primary hair follicles in mice [28], and the proliferation of primary hair follicle cells in KO mice at the E14 stage was inhibited, while apoptosis and oxidative stress signals were enhanced, and the morphology of primary hair follicles failed to form (Fig. 8f). Wild-type mice showed high expression of ncRNAs, and the PI3K-AKT-mTOR-ROS pathway was inhibited (Fig. 8e, f). At the P2 stage, clearly, visible thick primary hair follicles could be observed (Fig. 8f). This confirms the inhibitory effect of *Gtl2* (*Meg3*)-miRNAs on the PI3K-AKT-mTOR pathway and its importance in the development of primary hair follicles.

### Inhibiting the PI3K-mTOR-ROS pathway reduces oxidative stress in wool follicle stem cells, facilitating robust growth of primary wool follicles in ALC lambs

Separate analysis of proteomic down-regulated proteins enriched in metabolic pathways identified a range of signaling pathways associated with mitochondrial and energy metabolism (Additional file 2: Table S4). Metabolomic analysis of skin tissues also showed that the ALC group down-regulated metabolites were enriched in pathways related to mitochondria and energy metabolism (Additional file 1: Fig. S3). Comparisons using the orthogonal partial least squares analysis (OPLS-DA) method showed that the ALC and MF groups could be completely separated (Fig. 6f). Among the differential metabolites, two pairs of top metabolites were selected as candidate biomarkers, one pair being (nicotinamide adenine dinucleotide)/nicotinamide adenine dinucleotide) NAD+/NADH, which are cofactors that power ATP production in mitochondria and represent energy metabolism levels [32]. The other pair was glutathione (GSSG)/glutathione (GSH), which are recognized markers of oxidative stress [33]. The results showed higher NADH levels and lower NAD+ levels in the MF group, indicating a stronger oxidative phosphorylation state in the skin tissue of MF lambs (Fig. 6f). higher GSSG levels and lower GSH levels in the MF group further indicated a stronger oxidative stress state in the skin tissue of MF wool-type lambs (Fig. 6f). Interestingly, the high expression of the oxidative stress marker proteins COX2 [34] and glutathione peroxidyl/transferase in the MF group, again suggesting a strong oxidative stress state in the skin of MF lambs (Fig. 6d, Additional file 1: Figs. S3, S8). Combined miRNA-seq, proteomics and metabolomics analyses suggest that it may be that *Gtl2*-miRNAs act on PI3K-AKT-mTOR and mitochondrial energy metabolic pathways, which in turn cause redox imbalance in downstream targets, playing an important role in early wool follicle development. And they suggest a deficit of adaptation in MF fine wool lambs.

**Fig. 9:**
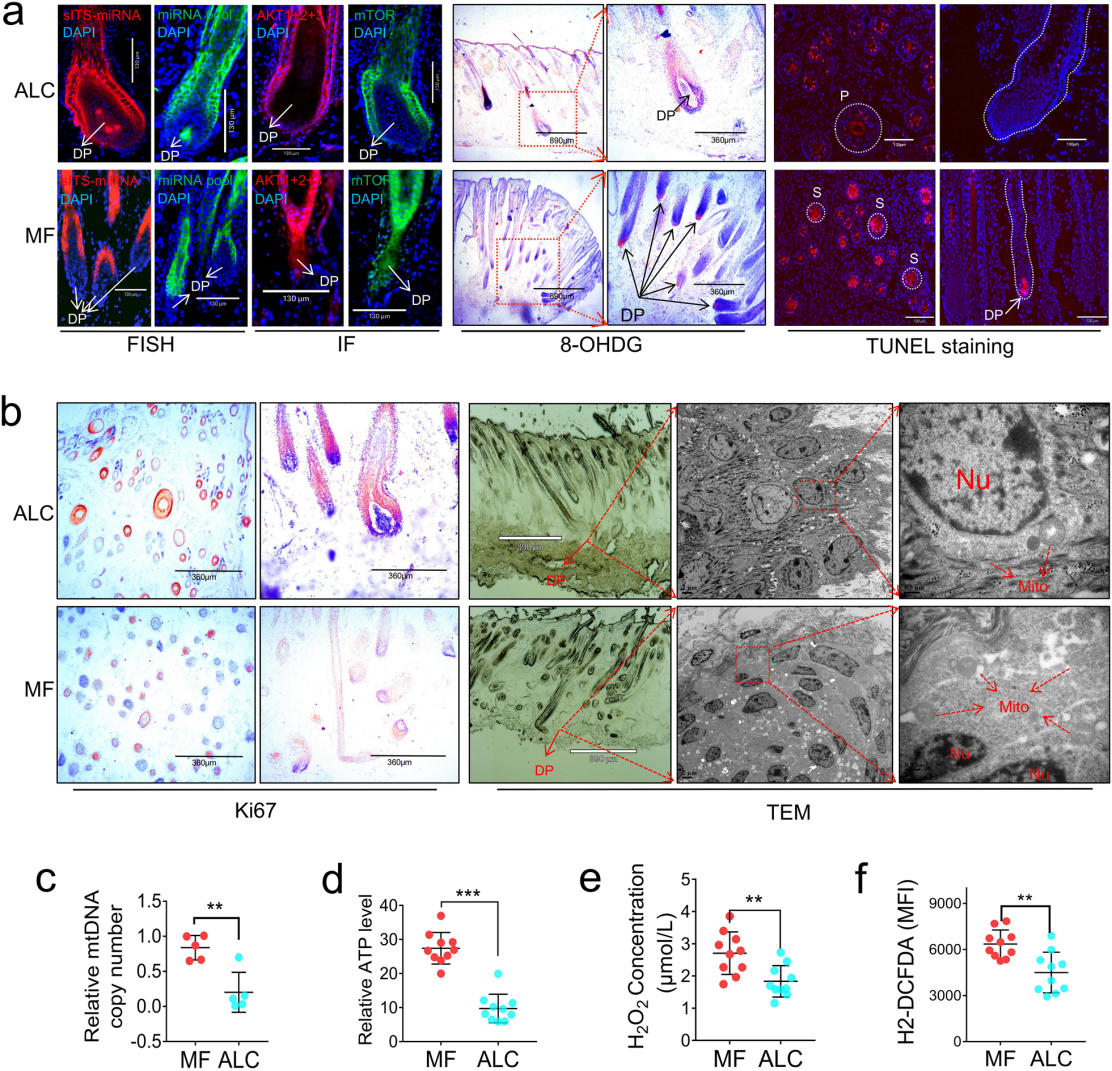
Redox imbalance leads to terminal differentiation, aging, and apoptosis of wool follicles in MF wool lambs. **a** The left: Activity of the PI3K/AKT signaling pathway in longitudinal sections of *Meg3* (IG-DMR)-KO mouse skin tissue as determined using immunofluorescence. The letter DP indicates dermal papilla. Middle: detection of the ROS level of 1-d reciprocal cross progeny between ALC and MF using 8-OHDG staining. The black arrow indicates the dermal papilla cell. Right: detection of skin cell apoptosis of 1-d reciprocal cross progeny between ALC and MF using TUNEL staining. **b** The left: detection of skin cell proliferation of 1-d reciprocal cross progeny between ALC and MF using Ki67 immunostaining. Right: skin section observations of 1-d reciprocal cross progeny by using transmission electron microscopy. The red dotted line indicates the position of dermal papillae, DP: dermal papilla, Nu: cell nucleus, Mito: mitochondria. **c** Detection of mitochondria DNA copy numbers in the skin tissue of 1-d reciprocal cross progeny between ALC and MF lambs (n = 5; **P < 0.01). **d** Detection of ATP levels in the skin tissue of 1-d reciprocal cross progeny between ALC and MF lambs (n = 10; ***P < 0.001). **e** Detection of H_2_O_2_ levels in the skin tissue of 1-d reciprocal cross progeny between ALC and MF lambs (n = 10; **P < 0.01). **f** Detection of reactive oxygen species (ROS) levels in the skin tissue of 1-d reciprocal cross progeny between ALC and MF lambs (n = 10; **P < 0.01)

The difference in energy metabolism between ALC and MF wool lambs suggest the possibility of a difference in mitochondrial function. The analysis revealed that the MF group had higher mitochondrial DNA copy number and ATP levels (Fig. 9c, d). At the same time, H_2_O_2_ levels were significantly higher in the skin of MF wool lambs (Fig. 9e) and ROS levels were significantly higher than those in the ALC group using H2-DCFDA and 8-OHDG staining (Fig. 9f, a, middle). The results of the oxidative stress experiment echoed the high levels of mitochondria-encoded COX2 (oxidative stress marker) and oxidative phosphorylation status in the MF group in the previous proteomic and metabolomic results (Fig. 6d, f, Additional file 1: Fig. S3). Taken together, these results suggest that low *Gtl2*-miRNA expression leads to activation of the PI3K-AKT-mTOR mitochondrial pathway, which in turn leads to accumulation of ROS in wool follicle stem cells, ultimately resulting in restricted wool follicle growth in MF wool lambs, maintaining smaller wool bulbs and finer wool fibers (Fig. 6e). In contrast, high expression of *Gtl2*-miRNA in ALC lambs inhibited this pathway, producing coarser primary wool follicles and coarser medullated wool fibers. Reduced oxidative stress in wool follicle dermal papilla cells underlies the vigorous growth of primary wool follicles in ALC lambs.

## Discussion

The ALC wool type sheep have their origins in the fine wool sheep population. The ALC wool type is initially observed during the birth and infancy stages of fine wool sheep, which later transitions to the same fine wool across the entire body, resembling the MF wool type sheep. Through SNP analysis of mRNA transcriptome, we located the ALC wool type trait to the *Gtl2*-miRNAs locus on chromosome 18. The *Dlk1*–*Gtl2* imprinting region plays a crucial role as an epigenetic regulatory locus that regulates embryonic and early developmental traits [7]. In domesticated animals, the *Callipyge* phenotype is determined by this locus [35]. The relationship between the *Gtl2*-miRNAs site and hair follicle development has not been reported. Previous studies have revealed that the *IRF2BP2* gene is the major candidate gene for sheep’s coarse and fine wool types [36, 37]. Genomic variations in the *IRF2BP2* locus have been used to distinguish between coarse and fine wool sheep breeds [37]. Therefore, in this study, we examined the *IRF2BP2* gene variation in the progeny of reciprocal cross test using normal Merino fine wool sheep and ALC wool type sheep. The genotype of both parents and offspring adhered to Mendelian inheritance patterns. However, we observed that the phenotype of the hybrid offspring consistently resembled that of the female parent, irrespective of the type of *IRF2BP2* gene variation (Additional file 1: Fig. S4). This indicates that the epigenetic effect discovered in this study is an independent regulatory mode that is separate from genomic variation, and the presence or absence of the ALC wool type is determined by the expression of non-coding RNAs at the imprinted *Gtl2*-miRNAs site. Furthermore, we discovered novel *Gtl2*-sITSs markers in the *Gtl2* gene exon, representing the first known s-ITSs sequence discovered in sheep. Among offspring produced from our reciprocal crosses test, the genotype and phenotype of the *Gtl2*-sITSs markers remained consistent with the female parent (the female parent is homozygote and covered with an ALC wool, whereas the male parent is heterozygous and has a MF wool type), suggesting that these *Gtl2*-sITSs related markers have an altered imprinted pattern. By using the NCBI's alignment search tool (BLAST), we examined the similarity of *Gtl2*-sITSs sequences across different species (Additional file 1: Fig. S2a) and found that *Gtl2*-sITSs are exclusively present in ruminants and marine mammals. Further analysis revealed that the earlier homologous sequences of this s-ITSs originated from bony fish (Additional file 1: Fig. S2a, c). The similarity of the *Gtl2*-sITS sequences among distantly related species suggests that this genetic feature may have been preserved from ancestral ruminants, potentially contributing to a unique regulatory mechanism specific to ruminants. This pattern extends to Tan sheep, a Chinese local breed of sheep known for its high-quality lambs fur. By re-analyzing publicly available miRNA sequencing data, we identified that the exceptional lamb fur quality observed in newborn Tan sheep is determined by primary wool follicles and regulated by the same imprinted *Gtl2*-miRNAs locus as ALC lambs (Additional file 1: Fig. S7a–c) [38].

The ALC wool lambs discovered in this study are not just about variations in wool type, but more importantly, they have increased body weights (Fig. 1n), enhanced wool yields and better wool quality (Fig. 1l, m), as they matured compared to MF lambs. This means that ALC wool sheep have higher body weight and wool production performance than MF wool sheep. In other words, they have some breeding advantages. Our results show that ALC lambs inhibit the PI3K-AKT-mTOR-ROS pathway systematically through the high expression of miRNAs at the *Gtl2*-miRNAs locus, reduce the ROS level of wool follicle stem cells, and keep them in a weaker oxidative stress state (Fig. 9a, e, f), indicating that ALC lambs have better adaptability. In contrast, popular MF wool-type lambs in breeding are clearly in a more stressful state, with extremely short, soft, and curly MF wool at birth, exposing their skin more to wet and cold conditions. The high death rate and high care cost of fine wool lambs in cold and harsh environments are apparently related to their small body size [18, 19], and the results of this study suggest that intense oxidative stress caused by their metabolism may be the more fundamental reason. This cold stress resistance trait and molecular mechanism may be the reason for the larger body weight of young ALC lambs (Additional file 1: Fig. S7d), which has also been demonstrated in *Meg3*(*Gtl2*)-DMR-KO mice (Additional file 1: Fig. S5). Extending the experimental population to other coarse and fine wool breeds sheep, we also found higher levels of ROS in the skin tissue and even in wool follicle papilla cells of young fine wool lambs compared to coarse wool breeds (Additional file 1: Fig. S7e–g). However, the age-dependent phenotype of ALC wool traits and the features based on epigenetics are easy to neglect and not utilized in conventional breeding and genomic selection. This also reflects the shortcomings of relying solely on genomic markers. Some traits must be identified and utilized by combining epigenetic information and specific imprinted modes [39]. This suggests that dual selection of genomics and epigenetics can provide a possible solution to the contradiction between yield and quality, and yield and adaptability.

ALC wool-type lambs not only exist in our experimental population, but according to informal surveys, they are also frequently found in the fine wool sheep populations in Xinjiang and Gansu provinces of China. However, these lambs are excluded from the breeding population when selected for breeding purposes. A similar situation has been observed in Australia, a renowned center for Merino sheep breeding and production. Dr. Neville Jackson [20] (of CSIRO Division of Animal Production, Prospect, NSW 2148, Australia) has initiated a global project to address the recurring incidence of individuals displaying primitive sheep characteristics, such as coarse wool resembling ancestral traits, within Australian Merino sheep flocks (https://github.com/nevillejackson/atavistic-sheep). According to history of the breed, Merino originated in Spain back to the fifteenth to seventeenth centuries, and then, Australia established its own thriving sheep industry by introducing Merino breeds from various other countries [40]. Therefore, our speculation is that the presence of a comparable ancestral wool type within the Australian Merino population may be attributed to the initial use of a local coarse female ancestor. However, the significance of this ancestral coarse wool type was overlooked during subsequent breeding efforts, resulting in its sporadic manifestation. These occurrences have defied explanation through conventional quantitative genetics theory, and no specific purpose for their utilization has been identified. Nevertheless, this study proposes that *Gtl2*-miRNAs imprinted genetics could provide an explanation for this phenomenon and maternal genetic effect could be exploited in future selective breeding.

The high expression of the *Gtl2*-miRNAs cluster in ALC lamb skin tissue has a favorable effect on meat and wool production. The reason for the high expression of *Gtl2*-miRNAs still needs to be further explored. We have recently found a correlation between the *POT1* gene and ALC traits by integrating transcriptome data and WGBS data [41]. Taking into account the function of *POT1* as a telomere protector [42, 43], we speculate that it could potentially influence the copy number of *Gtl2*-sITSs, which is a repetitive sequence with telomere sequence features. The relevant mechanisms need to be further studied.

## Conclusions

By comparing the ALC wool-type model with its genetically matched MF wool-type siblings, we investigated the epigenetic regulatory mechanisms involved in early wool follicle development. We discovered that the ALC wool type of Merino sheep is regulated by epigenetic mechanisms in the *Gtl2*-miRNAs imprinted gene cluster on sheep chromosome 18, with the maternal expressed imprinted gene in this locus responsible for the ALC phenotype. We confirmed that a novel 38-nt small RNA transcribed from a newly identified s-ITSs, in combination with the previously reported 22-nt small RNAs cluster from the *Gtl2*-miRNAs locus, synergistically inhibited PI3K/AKT/metabolic/oxidative stress and subsequent apoptotic pathways in wool follicle stem cells, resulting in the ALC wool type. The necessity of *Gtl2*-miRNAs in controlling primary hair follicle morphogenesis was verified by a knockout mouse model. We highlighted the potential challenges associated with contemporary molecular breeding theories and put forth a selection paradigm that emphasizes early trait selection to enhance animal adaptability and overall performance. This approach holds promise as an effective strategy to reconcile the trade-off between yield and adaptability in the future.

## Methods

### Sheep and mice

Chinese Merino ALC wool and MF wool lambs were provided by Jinfeng Animal Husbandry Group Co., Ltd (Chifeng, China). The skin samples from ALC and MF wool lambs were used in whole transcriptome (n = 8), proteome (n = 6), and metabolome (n = 10) analyses as well as in subsequent validation analyses (Additional file 2: Table S1). The lambs provided were half-siblings. Furthermore, 1-day-old lambskin samples of reciprocal cross progenies were used for TEM analyses (n = 6), TUNEL staining (n = 6), and ki67 (n = 6) and 8-OHdG immunohistochemistry (n = 6). Ten ALC wool lambs, five from the Chinese Merino population and five from the reciprocal cross progenies, were used for ATP testing, mitochondrial DNA (mtDNA) copy number analysis, western blotting, qPCR, mass spectrum identification, H_2_O_2_ and ROS level detection, and weight determination. The information of sheep samples for genotypic identification is as follows. ALC wool lambs (n = 5), MF wool lambs (n = 5), Australian Merino (n = 60), Aohan fine wool sheep (n = 50), and Ujimqin sheep (n = 37) were also provided by Jinfeng Animal Husbandry Group Co. Gansu alpine Merinos (n = 88) were purchased from Huangcheng sheep farm (Huangcheng, Gansu, China). The blood samples of Tan sheep (n = 39) and Small Tail Han sheep (n = 30) were provided graciously by Dr. Xinhai Li (College of Agriculture, Ningxia University, Yinchuan, China). The skin samples and wool fibers of Tan sheep (n = 10) were provided by Dr. Qing Ma (Animal Science Institute of Ningxia Agriculture and Forestry Academy, Yinchuan, China). Clustered Regularly Interspaced Short Palindromic Repeats-(CRISPR) associated protein 9 (CRISPR-Cas9)-mediated genome engineering was used to generate and breed *Meg3* (IG-DMR region included, 4675-bp nucleotide deletion)-KO mice with a C57BL/6J background targeting the *Meg3*-207(ENSMUST00000143836.7) transcript (VivoCure Science & Technology Co., Ltd., Beijing, China). Integration of the transgene was performed using PCR analysis, and tail tissues were used for DNA extraction. We strictly adhered to the Guide for the Care and Use of Laboratory Animals in China. All experiments were approved by the Committee on the Ethics of Animal Experiments of China Agricultural University (permit number: SKLAB-2012-04-07).

### Sheep wool, skin, and mouse skin

The wool fibers of ALC, MF, and Tan sheep in middle-sided dorsal skins (P30, P120, and P180) were harvested for observation and determination of wool characteristics. ALC and MF middle-sided dorsal skins at P1, P30, and P180 were quickly collected and frozen in liquid nitrogen for total RNA extraction, protein extraction, and frozen sections. The freshly collected skin tissues were fixed with glutaraldehyde (2.5%) at 4 °C overnight for TEM analysis. The *Meg3* (IG-DMR)-KO mouse dorsal skin tissues at E14, P1, and P2 were quickly collected and frozen in liquid nitrogen for total RNA extraction, protein extraction, and frozen sectioning. The KO mice and their negative controls in this study were all females. The mice in each experiment were full sibs.

### Collection of sheep body weight and fleece yield

The weight at 1 month of age was recorded as lamb weight and the weight at 12 months of age as adult weight. At yearling shearing, the grease fleece was weighed. The comparisons of body weight and grease fleece between the two groups was conducted using two-tailed *t*-tests, and the p-values were as follows: *p < 0.05; **p < 0.01; ***p < 0.001.

### Observation and determination of wool characters

The wool diameter was measured using a microscope projector (BEINO M1, Shanghai, China). Three pairs of half-sib ALC and MF, as well as six Tan sheep were used to determine wool diameter using a Y172 fiber slicer (Hastelloy slicer), each of which included 1000 fibers, and 500 fibers of Tan sheep were also used to test the wool diameter. The measured results were recorded sequentially, and the average fiber diameter was calculated to determine the fiber diameter distribution and the proportions of different wool types.

### RNA isolation

Total RNA was isolated from the sheep and mouse skin using TRIzol reagent (Thermo Fisher Scientific, MA, USA). RNA contamination and degradation were visualized using agarose gel (1%). RNA purity was performed using a NanoPhotometer spectrophotometer (IMPLEN, CA, USA), and the RNA concentrations were determined using the Qubit RNA Assay Kit in Qubit 2.0 Fluorometer (Life Technologies, CA, USA). RNA integrity was checked using the RNA Nano 6000 Assay Kit of the Bioanalyzer 2100 system (Agilent Technologies, CA, USA). The total extracted RNA was used for subsequent RNA-seq and qPCR experiments.

### Detection of RNA-seq variants

The quality of the sequencing data was determined using FastQC (https://www.bioinformatics.babraham.ac.uk/projects/fastqc/), whereas FASTP was used to conduct quality control of sequencing data using default parameters (https://github.com/OpenGene/fastp). Sequencing reads of RNA-seq were aligned to the sheep reference genome (GCA_000298735.2 and GCA_016772045.2, NCBI) using the STAR algorithm with default parameters. Subsequently, PCR duplicates were removed using the MarkDuplicates module in the Picard Tools package (http://broadinstitute.github.io/picard/). The GATK 4.0 module HaplotypeCaller (https://github.com/broadinstitute/gatk) was used to conduct variant calling, and variant filtering was performed using the parameters “QUAL < 30, QD < 2.0, MQ < 40.0.”

### MicroRNA-seq, lncRNA-seq, and mRNA-seq

A total of 4 μg of RNA from each sample was used as input material for RNA sample preparation. Ribosomal RNA was removed using the Epicentre Ribo-zero™ rRNA Removal Kit (Epicentre, WI, USA); the lncRNA and mRNA sequencing libraries were constructed using the NEBNext® Ultra™Directional RNA Library Prep Kit for Illumina® (NEB, MA, USA), according to the manufacturer’s instructions. The microRNA libraries were constructed using the NEBNext Multiplex Small RNA Library Prep Kit for Illumina® (NEB, MA, USA), and the index codes were used to attribute the sequences to each sample. Clustering of the index-coded samples was performed on the cBot Cluster Generation system using the TruSeq PE Cluster Kit v3-cBot-HS (Illumina). Subsequently, the mRNA and lncRNA libraries were sequenced on the Illumina Hiseq 2000 platform. The microRNA libraries were performed using the Illumina Hiseq 2500/2000 platform. The sheep reference genome and annotation files were obtained from the Ovis genome website (ftp://ftp.ncbi.nlm.nih.gov/genomes/all/GCF_000298735.2_Oar_v4.0/GCF_000298735.2_Oar_v4.0_genomic.fna.gz). The clean reads were obtained from raw reads by removing poly-N regions, reads containing adapters, and low-quality reads (Trimmomatic). The Q20, Q30, and GC contents of the clean reads were calculated. The reference genome index was constructed using Bowtie2, and clean paired-end reads were aligned to the reference genome using TopHat. The mapped reads of each sample were assembled using both Cufflinks and Scripture (beta2) with a reference-based approach. The transcripts were either predicted based on the coding potential by the Coding-Non-Coding-Index, Coding Potential Calculator, 0.9-r2 (CPC), or Pfam Scan, and the phylogenetic codon substitution frequency (PhyloCSF) and all four programs were filtered out. Subsequently, those without coding potential were designated as the candidate lncRNA set. We used the PhyloFit program to compute phylogenetic models for conserved and non-conserved regions among species. The phastCons program was used in conjunction with the model and HMM transition parameters to compute a set of conservation scores for the mRNAs and lncRNAs. The Cuffdiff algorithm was used to calculate the fragments per kilobase of exon per million fragments mapped (FPKMs) of both lncRNAs and mRNAs in each sample. Using a model based on the negative binomial distribution, the DEGs with an adjusted p-value (p-adjust < 0.05; Benjamini–Hochberg multiple test correction) between ALC and MF sheep were identified.

### Proteome analysis

TMT-proteome analysis was performed at Bioprofile, Shanghai, China. To lyse the sheep skin tissues (samples similar to MicroRNA-seq, lncRNA-seq, and mRNA-seq), they were suspended on ice in 200 μL radioimmunoprecipitation assay lysis buffer (4% SDS, 100 mM DTT, 150 mM Tris-HCl pH 8.0). The tissues were then disrupted with agitation using a homogenizer and boiled for 10 min. The samples were further ultrasonicated and boiled for another 5 min. Undissolved cellular debris was removed by centrifugation at 16,000 rpm for 20 min. The supernatants were collected and quantified using a BCA protein assay kit (Beyotime, China). Protein analysis of each sample (200 μg) was performed. Briefly, the detergent, DTT, and other low-molecular-weight components were removed using 200 μL UA buffer (8 M urea, 150 mM Tris-HCl pH 8.0). Then, 100 μL 0.05 M iodoacetamide in UA buffer was added to block reduced cysteine residues, and the samples were incubated for 20 min in darkness. The filter was washed with 100 μL UA buffer three times and then 100 μL 25 mM NH_4_HCO_3_ twice. Finally, the protein suspension was digested with 4 μg trypsin in 40 μL 25 mM NH_4_HCO_3_ overnight at 37 °C, and the resulting peptides were collected as a filtrate. The peptide concentration was determined with an OD280 using a Nanodrop device. The peptides were labeled with the TMT reagents (Thermo Fisher Scientific). After each sample was dissolved in 100 μL of 0.05 M TEAB solution, pH 8.5, the TMT reagent was dissolved in 41 μL of anhydrous acetonitrile. The mixture was incubated at 25 °C for 1 h. Then, 8 μL of 5% hydroxylamine was added to the sample and incubated for 15 min to quench the reaction. Multiplex labeled samples were pooled together and lyophilized. The TMT-labeled peptide mixture was fractionated using a Waters XBridge BEH130 column (C18, 3.5 μm, 2.1 × 150 mm) on an Agilent 1290 HPLC operating at 0.3 mL/min. The fractions were then dried for nano-LC–MS/MS analysis. The Shanghai Bioprofile company performed the LC–MS analysis on a Q Exactive HF-X mass spectrometer coupled to Easy nLC (Thermo Fisher Scientific). The resulting LC–MS/MS raw files were imported into Proteome Discoverer software (version 2.4) for data interpretation and protein identification against the database Uniprot_Ovis aries (Sheep)_28192_20201116.fasta (downloaded on 16/11/2020, including 28,192 protein sequences), which is sourced from the protein database (https://www.uniprot.org/uniprot/?query=Ovis-aries&sort=score). Analyses of bioinformatics data were carried out using Perseus software, Microsoft Excel, and R statistical computing software. Significant DEPs were screened with the cutoff ratio Log_2_^Foldchange^ < − 0.32 or > 0.26 and p-values < 0.05. Protein hierarchical clustering was used to group the expression data together. To annotate the sequences, information was extracted from UniProtKB/Swiss-Prot. GO and KEGG enrichment analyses were implemented using DAVID Bioinformatics Resources 6.8 (https://david.ncifcrf.gov/) with a corrected p-value cutoff of 0.05 to judge statistically significant enrichment.

### PRM analysis

In order to verify the protein expression levels obtained by TMT-proteome analysis, the expression levels of 21 selected proteins were quantified using LC-PRM/MS. After the peptides were prepared using the TMT protocol, tryptic peptides were loaded on C18 stage tips on an Easy nLC-1200 system (Thermo Scientific). One-hour liquid chromatography gradients (acetonitrile ranging from 5 to 35% in 45 min) were performed. The Q Exactive Plus mass spectrometer (Thermo Scientific) was used for PRM analysis. Full MS1 scans were obtained with the spectrometer. In brief, there were 20 PRM scans at 35,000 resolution (at m/z 200), automatic gain control (AGC) 3.0 × 106, and maximum injection time of 200 ms. The targeted peptides were isolated (2Th window) and fragmented at a normalized collision energy of 27 in a higher energy dissociation (HCD) collision cell. Skyline (MacCoss Lab, University of Washington) was used to analyze the raw data, and the signal intensities of the peptide sequences were acquired for subsequent analysis. For PRM-MS data of the ALC and MF groups, rawMeat (version 2.1, VAST Scientific, www.vastscientific.com) was used to extract the sample average base peak intensity (F_N_). Furthermore, N/the median of average base peak intensities of all samples was used to calculate the normalization factor for sample N. This factor was multiplied by the area under curve (AUC) for each transition from sample N. Then, the AUC of each transition was summed to obtain the AUCs at the peptide level. The intensity of certain peptides was identified as relative to the protein’s abundance.

### Metabolome analysis

Metabolomic analysis was performed by Bioprofile in Shanghai, China. The ALC and MF lambskin (P1) samples were weighed, and 100 mg of the samples were transferred to Eppendorf tubes (1.5 mL). Two small steel balls were added to the tubes with 600 μL extraction solvent plus methanol/water and 20 μL internal standard, and then the tubes were stored at − 80 °C for 2 min. Subsequently, the samples were ground at 60 Hz for 2 min. Chloroform (120 μL) was added to the tubes. Then the tubes were vortexed, subjected to a 10 min ultrasound extraction, and stored for 10 min at 4 °C. Subsequently, the tubes were centrifuged at 4 °C for 10 min (12,000 rpm). Aliquots of all the samples were mixed and pooled to prepare QC. An aliquot of the 200 μL supernatant was vacuum-dried and transferred to a glass vial at room temperature. Next, 80 μL of methoxylamine hydrochloride was added to the mixture and vortexed for 2 min (37 °C for 90 min). Then, 20 μL *n*-hexane and 80 μL of BSTFA (1% TMCS) was added to the mixture, vortexed for 2 min, and derivatized for 60 min at 70 °C. After the samples were incubated at room temperature for 30 min, GC–MS was performed. The prepared samples were analyzed on the Agilent 7890B gas chromatography system coupled to an Agilent 5977A MSD system (Agilent Technologies Inc., CA, USA). The ion source and quadrupole were performed at 150 and 230 °C, respectively. The collision energy was 70 eV. The results for mass spectrometric analysis were available at m/z 50–500. The raw data was analyzed using AnalysisBaseFileConverter software. The LUG database was used to annotate metabolites. Total peaks were detected from all samples. The known pseudo positive and standard peaks were also removed. An orthogonal partial least-squares-discriminant analysis (OPLS-DA) was performed to reveal the metabolic difference between ALC and MF lambs. The group discrimination was performed through variables with VIP > 1. The statistically significant threshold for differential metabolites was VIP values larger than 1.0 and p-values less than 0.05.

### Functional verification of candidate miRNAs

sFFCs were successfully isolated in our lab [44]. We obtained HEK293T cells from the Institute of Biochemistry and Cell Biology, Chinese Academy of Science. The sFFCs and HEK293T cells were cultured in Dulbecco’s modified Eagle’s medium with 10% fetal bovine serum and streptomycin/penicillin. The cells were cultured at 37 °C with 5% CO_2_. The sFFCs and HEK293T cells were used to validate the candidate miRNA target and miRNA function. HEK293T cells were inoculated into 24-well plates (Corning Incorporated, US). *Oar-miR-668-5p* was co-transfected with 150 ng of psicheck2-candidate gene (*Gnb1*, *Pir3r1*, *Akt1*, *Akt2*, *Akt3*, *mTOR*, *Rheb*, *Rac1*, *Itgav*, *Col6a2*, *Atp6v1a*, *Atp6v1c2*, *Atp6v1b2*, *Atp6v1f*, *Atp6v1g1*, *Atp6v1h*, *Mecr*, *Aco1,* and *Bdh1*)-fragments and 60 pmol of *oar-miR-668-5p* mimics, and the negative control mimics were RiboBio (Guangzhou, China) with lipofectamine 2000 (Invitrogen, USA). Furthermore, *sITS*-miRNA mimics was co-transfected with 150 ng of psicheck2-candidate gene (*Gnb1*, *Pir3r1*, *Akt1*, *Akt2*, *Rela*, *mTOR*, *Nfkb1*, *Rac1*, *Itgav*, *Col6a2*, *Atp6v1a*, *Atp6v1c2*, *Atp6v1b2*, *Atp6v1f*, *Atp6ap1*, *Mecr*, *Aco1*, *Bdh1*, and *Cox5a*)-fragments and 50 pmol of *sITS*-miRNA mimics, and the negative control was RiboBio (Guangzhou, China). Forty hours after transfection, renilla and firefly luciferase activities were tested using a dual luciferase reporter kit (Promega, USA). Each assay was repeated three times. For the functional verification of candidate miRNAs (*Oar-miR-668-5p*, *Oar-miR-412-3p*, *Oar-miR-541-3p*, *Oar-miR-323b*, *Oar-miR-544-5p*, *Oar-miR-154a-3p*, and *Oar-miR-136*), the sFFCs were inoculated into 12-well plates, and 24 h later, the microRNAs pool (*Oar-miR-668-5p*, *Oar-miR-412-3p*, *Oar-miR-541-3p*, *Oar-miR-323b*, *Oar-miR-544-5p*, *Oar-miR-154a-3p*, and *Oar-miR-136*) mimics, negative control, and inhibitors were transfected with lipofectamine 2000 (Invitrogen, USA). The cells were collected 48 h after transfection for further analysis (qPCR and western blot). Additionally, *sITS*-miRNA mimics and the negative control were transfected into the sFFCs using six-well plates for 60 h. The cells from each well were collected for total protein and RNA extraction, which were used in subsequent qPCR and western blot assays.

### TEM analysis

Fresh sheep skin tissues were fixed with 2.5% glutaraldehyde at 4 °C overnight. The skin tissues were then washed six times with phosphate-buffered saline (PBS; 0.1 M), fixed with OsO_4_ (1%) at 4 °C for 1 h, and washed three times again with 0.1 M PBS. An ethanol gradient series (30% for 15 min, 50% for 15 min, 70% for 15 min, 80% for 15 min, 90% for 15 min, 95% for 15 min, and 100% for 30 min) was used for dehydration. Acetone/ethanol at a volume ratio of 1:1 was used for overnight infiltration. The next morning, different ratios of acetone/embedding medium (2:1, 3 h; 1:1, 4 h; 1:2, 4 h) were used for infiltration, and then absolute embedding medium was used for overnight infiltration. The following temperature series, 37 °C for 12 h, 45 °C for 12 h, and 60 °C for 48 h, was used for polymerization. A Leica ultramicrotome (Leica EM UC7, Germany) was then used to slice 50-nm skin sections. The sections were stained with lead citrate and then photographed under TEM (JEM-2010F, JEOL Ltd., Japan).

### TUNEL staining

TUNEL staining was performed according to the manufacturer’s instructions (Beyotime, C1090, Shanghai, China). Frozen 8-μm-thick sections were prepared using a freezing microtome (Leica CM1900, Germany). The sections were then fixed with 4% paraformaldehyde for 60 min. After being washed twice with PBS (10 min each time), the cells were incubated in PBS with 0.5% Triton X-100 (5 min, room temperature). The sections were washed twice again with PBS, and then 50 μL prepared TUNEL working solution with two components (5 μL TdT enzyme, 45 μL fluorophore-labeled solution) was used to incubate the sections with light at 37 °C for 60 min. Subsequently, the sections were washed three times with PBS. The anti-fluorescence quenching sealing solution was used to seal the sections, and photographs were taken using a fluorescence microscope (ECHO, RVL-100-G, USA).

### ATP level assessment

ATP levels of ALC and MF skin were measured using an ATP testing assay kit (Beyotime, S0027, China). Briefly, the prepared frozen sections were prepared as described previously. Fresh skin tissues were sufficiently ground in a mortar with liquid nitrogen, and the ground tissues were then lysed in an ATP lysis solution for 10 min and centrifuged at 12,000 rpm for 10 min. The supernatants were obtained and mixed with a testing buffer. The concentration of ATP in each sample was measured using a luminescence detector (TECAN, Infinite M200, Switzerland). The experiments were divided into two groups (ALC and MF), and each group contained 10 samples.

### MtDNA copy numbers

Total DNA of ALC and MF skin was extracted by incubating in lysate solution (0.5% Tween-20, 50 mM Tris-HCl, 100 mg/mL protease K, and 1 mM EDTA) for 10 h at 55 °C. For full lysis, the products were heated to 90 °C for 30 min. The samples were then subjected to qPCR with primers nd1-f1 and nd1-r1 (Additional file 2: Table S5). The copy numbers of mtDNA were normalized to those of β-actin (Additional file 2: Table S5). The qPCR was performed using SYBR premix (Tiangen Biotech, Beijing, China) according to the manufacturer’s instructions.

### Measurement of H_2_O_2_ levels

The fresh skin tissues of ALC and MF were cut up fully and mixed with the lysis buffer solution in the hydrogen peroxide assay kit (Beyotime, Jiangsu, China; 100 mL per 5 mg). The following tests were performed by gathering the supernatants and centrifuging them at 12,000 × rpm for 10 min. All steps took place on ice. Next, the tubes containing the 50 mL supernatant and 100 mL test solution were placed at room temperature for 30 min, and the absorbance at 560 nm was immediately measured using a microplate reader (DTX880, Beckman). The levels of H_2_O_2_ in ALC and MF skin tissues were calculated through a standard concentration curve based on the kit’s protocol.

### Measurement of ROS levels

The ROS levels were measured using a 2′,7′-dichlorodihydro-fluorescein diacetate kit (E-BC-K138-F, Elabscience, USA). In brief, the fresh skin tissue from the ALC and MF wool lambs’ dorsal skin were collected for the preparation of a single cell suspension by the enzymatic isolation method. The collected tissue was quickly added to buffer solution 3 and then fully cut up. Then, the fragments of tissue were removed from buffer solution 3. Subsequently, enzymes were added and incubated overnight to digest. The next morning, medium containing serum was added to stop the enzyme digestion. Next, the single cells were resuspended in buffer solution 3. Then, the single cells were added to a working solution (reagent 1). The reaction was then kept away from light and incubated for 30 min at 37 °C. Subsequently, cells were collected after centrifugation for 5 min (5000 × rpm) and washed three times with working solution 3. The cell precipitate was suspended in buffer solution 3 and analyzed with a microplate reader (DTX880, Beckman). Resuspended cells were used for measuring the fluorescence value at the excitation and emission wavelengths of 500 nm and 525 nm. Three parallel replicates were set for each sample.

### 8-Hydroxy-2′-deoxyguanosine (8-OHdG) staining

Fresh skin tissues of ALC and MF were fixed with 4% formaldehyde and then embedded in paraffin. They were then cut fully, and immunohistochemical was performed using 5 μm-thick sections. Skin samples were stained with the immunoperoxidase affinity biotin method using an anti-8-hydroxy-2′-deoxyguanosine antibody (N45.1, ab48508, Abcam, USA, 1:50) overnight at 4 °C followed by an HRP-conjugated Goat anti-mouse IgG as a secondary antibody for 1.5 h. The antigen–antibody reaction was observed using 3,3-diaminobenzidine tetrahydrochloride (DAB) by incubating for 5 min at room temperature. Next, an alcohol gradient was used for dehydration, and neutral gum was used to seal the slices. Images were taken under a light microscope (ECHO, RVL-100-G, USA).

### *MEG3*(IG-DMR)-KO mouse

The IG-DMR in the *Meg3* (*Gtl2*)-miRNAs locus has been found and proven in mice [25]. This study aimed to delete this IG-DMR using the CRISPR/Cas9 system. Guide RNA vectors were designed and constructed, and the correct vectors were identified by gene sequencing. The Cas9 mRNA and guide RNA transcribed in vitro were introduced into the inbred strain mice zygote with C57BL/6J background by microinjection. The living mouse embryos after microinjection were transplanted into female recipient ICR mice. After 19 to 20 d of waiting, the progenies were born from recipient mice as F0. Genomic DNA of F0 was extracted from each mouse and used for genotype identification so that heterozygote mice with a chimeric genotype could be identified. Female heterozygote mice (approximately 2 months of age) of F0 were used to generate F1 mice by mating with C57BL/6J wild males. After PCR testing to identify *Meg3* (IG-DMR) mice, the F1 progeny were used for further experiments in this study.

### qPCR analysis

The mRNA expression levels of candidate coding and non-coding genes were validated by qPCR, which was performed using SYBR Premix ExTaq (Tiangen Biotech, Beijing, China) on the CF96 real-time system (Bio-Rad, USA). The cDNA synthesis of coding genes was performed using Quantscript RT Kit Quant cDNA (Tiangen Biotech, Beijing, China) with approximately 300 ng of total RNA as the template. For lncRNA and miRNA, the lnRcute lncRNA First-Strand cDNA Kit (Tiangen Biotech, Beijing) and the TaqMan® MicroRNA Reverse Transcription Kit (Thermo Fisher Scientific, USA) were used, respectively. SYBR Premix ExTaq (Tiangen, Beijing, China) was used for real-time PCR, which is available for an ABI7500 Real-Time PCR system (Applied Biosystems). The Tm values for each reaction are listed in Additional file 2: Table S5. The miRNA primers were designed and purchased from RiboBio Co., Ltd. (Guangzhou, China). The β-actin gene was used as a reference gene, and U6 was used as the miRNA reference control. Each plate was repeated three times in independent runs for all the references. Gene expression was evaluated using the 2^−∆∆Ct^ method [45].

### Western blot

The proteins of mouse skin, ALC and MF wool lamb skin, and sFFCs (transfected with miRNA mimics, NC, inhibitor, *sITS*-miRNA mimics, and *sITS*-miRNA NC) were extracted and electrophoresed in a 10% SDS/polyacrylamide gel (SDS-PAGE Gel Quick Preparation K) and transferred to a 0.45 μm Polyvinylidene fluoride, PVDF membrane. The blots were blocked in QuickBlock™ Blocking Buffer for western blot (Beyotime, Shanghai, China) for 1 h and probed with antibodies (for mouse skin western blot) phospho-PI3-kinase p85-alpha/gamma (Abmart, T40116F, China); anti-AKT1 + AKT2 + AKT3 (Abcam, ab179463, USA); mTOR antibody (Abmart, T55306, China); and β-actin antibody (Beyotime, AA128, China) in primary antibody dilution buffer (Beyotime, P0023A, China) overnight. The antibodies for sheep skin and sFFCs were: phospho-PI3-kinase p85-alpha (Tyr607) antibody (Affinity, AF3241, USA); anti-AKT (GeneTex, GTX128415, USA); and mTOR antibody (GeneTex, GTX101557, USA). Signals were detected using HRP-labeled secondary antibodies and a chemiluminescent substrate (BeyoECL Moon) (Beyotime, A0208 or A0216, China).

### Histology and immunohistochemistry

Sheep **(**ALC and MF) and mouse skin tissues were fixed in 4% paraformaldehyde formalin in PBS at 4 °C overnight, embedded in paraffin, sectioned at 5 μm, and stained with a hematoxylin and eosin staining kit (H&E). The following antibodies were used for immunostaining: anti-Ki67 (Abcam, ab15580), anti-Caspase 3 (Abcam, ab184787), anti-AKT1 + AKT2 + AKT3 (Abcam, ab179463); phospho-PI3-kinase p85-alpha/gamma (Abmart, T40116F); mTOR antibody (Abmart, T55306); HRP-labeled goat anti-rabbit IgG(H+L) (Beyotime, A0208), and HRP-labeled goat anti-mouse IgG(H+L) (Beyotime, A0216). The signal was detected using the DAB Horseradish Peroxidase Color Development Kit (Beyotime, P0202), and the sections were stained with hematoxylin. Photographs were taken using a microscope (ECHO, RVL-100-G, USA).

### Immunofluorescence

Frozen sections of skin tissue up to 6 µm thick were prepared and fixed in pre-chilled acetone for 10 min, then washed three times with PBS for 10 min each and incubated for 30 min at room temperature with 0.5% Trition100 (50 µL of trition100 in 100 ml of PBS). Trition incubation was not required for immunofluorescence experiments with mTOR antibody (Abmart, T55306) in this study. PBS was washed three times for 10 min each and blocked with 1% BSA for 1.5 h. The antibodies (anti-AKT1 + AKT2 + AKT3:Abcam, ab179463, phospho-PI3-kinase p85-alpha/gamma:Abmart, T40116F, mTOR antibody:Abmart, T55306) were diluted in BSA and incubated overnight in a wet box at 4 °C. The antibodies were recovered and the sections were washed 3 times with PBS for 10 min, added with a fluorescent secondary antibody corresponding to the primary antibody, incubated for 1 h at room temperature in a wet box, washed 3 times with PBS, added with DAPI and photographed under a microscope (ECHO, RVL-100-G, USA).

### Fluorescence in situ hybridization

The *sITS*-miRNA and miRNA pool (*Oar-miR-668-5p*, *Oar-miR-412-3p*, *Oar-miR-541-3p*, *Oar-miR-323b*, *Oar-miR-544-5p*, *Oar-miR-154a-3p*, and *Oar-miR-136*) probes were synthesized at GenePharma (Suzhou, China), and the specific fluorescent in situ hybridization is shown below. (1) Freeze sections were rehydrated, then remove frozen sections, add 100 μL of Buffer B (Citric acid buffer) dropwise to each section and leave for 15 min at room temperature. Aspirate Buffer B and wash the sections twice with PBS for 5 min each. (2) Proteinase K working solution pre-warmed to 37 °C. 100 μL of Proteinase K working solution was added dropwise to each section and incubated at 37 °C for 20 min. Each section was rinsed 3 times at room temperature with 2× Buffer C (20 × SSC) 100 μL dropwise, each time for 1 min. Gradient alcohol 70%, 80%, 90%, 100% dehydration, 2 min each time, dry in air Drying. (3) 78 °C preheated denaturing solution. Add 100 μL of preheated denaturing solution dropwise to each section and incubate for 8 min. Gradient alcohol 70%, 80%, 90%, 100% dehydration, 2 min each time, drying in air Drying. (4) Incubate Buffer E (Hybridisation buffer) in a water bath at 73 °C for 30 min until clear and bright. Dilution of the probe by adding 41.7 μL of sterilized DEPC water to each OD probe dry powder product. 4 μM probe stock solution was added to Buffer E (Hybridisation buffer). The total system was 100 μL and denatured for 5 min at 73 °C to prepare the probe mix. The sections were placed horizontally in a wet box and 100 μL of denatured probe mix was added dropwise to each section, coverslip and seal the slice with sealant. Incubate for 12–16 h at 37 °C in an in situ hybridiser, taking care to maintain the humidity to prevent the film from drying out. (5) 43 °C pre-heated washing solution. Gently remove the coverslip, aspirate the hybridization solution and add a drop of pre-warmed washing solution to each section 100 μL and wash the sections for 15 min at 43 °C. Each section was washed twice with 2× Buffer C (pre-warmed to 37 °C) in 100 μL drops, each time 10 min. PBS wash sections 1 time for 10 min. (6) Add 100 μL of diluted DAPI working solution to each section and incubate at room temperature and protected from light 10–20 min. Aspirate the DAPI working solution and wash the sections 2 times with PBS for 2 min each time. Add a drop of glycerol or anti-quenching agent, cover the slide and seal the slide with sealant under a fluorescent microscope (ECHO, RVL-100-G, USA) for observation.

### BLAST and phylogenetic analysis of interstitial telomeric sequences

A distance tree of the BLAST hits was performed using the NCBI BLAST service tool, which was used to retrieve and select matched sequences from the BLAST results. First, the short interstitial telomeric sequence (sITS) in *Gtl2* was submitted to the NCBI database BLAST (http://www.ncbi.nlm.nih.gov/BLAST). Next, a pseudo multiple sequence alignment (MSA) of the sITS and BLAST hit sequences was created by parsing the standard BLAST output. Finally, this MSA was turned to ClustalW, which produced a similarity tree (p-distance) by using the “-tree” option. This tree was built using the neighbor-joining method.

### Statistical analysis

All statistical details for the high-throughput sequencing experiments can be found in the above sections (MicroRNA-seq, lncRNA-seq, and mRNA-seq; Proteome analysis; and Metabolome analysis). In addition to the abovementioned analyses, PCA and volcano plots of the miRNA-seq, mRNA-seq, proteome, and metabolome data were performed using the R package (Version 4.2). Venn diagrams of DEGs and DEPs were performed using an online Venn diagram tool (http://bioinformatics.psb.ugent.be/webtools/Venn/). For all comparisons of independent observations between two groups, two-tailed *t*-tests were performed with the following p-values: *p < 0.05; **p < 0.01; ***p < 0.001.

### Supplementary Information

**Additional file 1: Figure S1.** Wool characteristics of ancestral-like coarse (ALC) and modern fine (MF) wool sheep at P120 and P180. (a) The phenotypic properties of ALC wool sheep at P120 and P180. (b) The phenotypic properties of MF wool sheep at P120 and P180. (c) Proportion of medullated and unmedullated wool fibers at different developmental stages in ALC sheep. (d) Proportion of medullated and unmedullated wool fibers at different developmental stages in MF sheep. **Figure S2.** Wool characteristics of ancestral-like coarse (ALC) and modern fine (MF) wool sheep at P120 and P180. (a) Phylogenetic tree showing the evolutionary relationships of Gtl2-sITSs in various species. (b) Percentage of heterozygotes and homozygotes between ALC wool and MF wool varieties. (c) A small fraction of sITS in different species. **Figure S3.** Pathway enrichment analysis performed using the significantly downregulated metabolites in ancestral-like coarse (ALC) lambskin tissue. **Figure S4.** *IRF2BP2* genotypes of ALC and MF wool lambs in reciprocal cross families. The primers were listed in Table S5. **Figure S5.** Embryonic weight of *Meg3*-IG-DMR-KO mice and their siblings (negative control), **P < 0.001. **Figure S6.** miRNAs in the *Gtl2*-miRNAs Locus inhibited multiple components of the PI3K-AKT Pathway. (a) The frequency of signaling pathways enriched by predicted target genes of up-regulated miRNAs in ALC group. (b) Schematic of the PI3K-mTOR pathway. (c) The up-regulated differentially expressed (DE) miRNAs at *Gtl2*-miRNAs locus and their predicted target genes in the PI3K-AKT pathway. **Figure S7.** Similar molecular mechanisms affect ALC wool traits and quality of lamb fur. **a** Curve graph of wool diameter distribution of Tan sheep. **b** Proportion of medullated and non-medullated wool of Tan sheep. **c** Integrated analysis of upregulated miRNAs and the functional annotation of their target genes between early developmental Tan and ALC lambs [38]. **d** Birth weight of ALC and MF wool lambs. **e** The skin ROS level of Coarse and Fine wool lambs were measured by H2-DCFDA, Coarse wool lambs: Ujimqin lambs (n = 30), Tan lambs (n = 10) and ALC wool lambs (n = 5). Fine wool lambs: Merino lambs (n = 60) and MF wool lambs (n = 5). **f** 8-OHdG immunostaining was perform in section of the Merino lamb skin. **g** The skin ROS level of adult Coarse/Fine wool sheep were measured by H2-DCFDA, Coarse wool sheep: Ujimqin sheep (n = 30), Tan sheep (n = 10) and ALC wool sheep (n = 5). Fine wool sheep: Merino sheep (n = 60) and MF wool sheep (n = 5). **Figure S8.** Interaction analysis of down-regulated DEPs in ALC group using online tool (https://www.string-db.org/). **Figure S9.** Selective signal analysis of transcriptome data using different reference genomes. **a** The samples are obtained from half-siblings, genome version: GCA_000298735.2, Oar_V4.0. The blue dashed line: Fst = 0.6. **b** The samples are obtained from the reciprocal cross progeny, and the genome version is GCA_016772045.2. The blue dashed line: Fst = 0.8.

**Additional file 2: Table S1.** The pedigree relationship between ALC and MF sheep in this study. **Table S2.** miRNAs in the Dlk-Gtl2 locus and their predicted target genes in the candidate pathway. **Table S3.** The predicted target genes of sITS-miRNA in the candidate pathway. **Table S4.** The separate enrichment analysis of the downregulated proteins in metabolic pathways of the ALC group. **Table S5.** Primers used in this study. **Table S6.** Guide RNA sequence information in *Meg3* (IG-DMR)-KO mice.

## Data Availability

The mRNA-seq, miRNA-seq, and lncRNA-seq data reported in this study have been deposited in the National Center for Biotechnology Information database with the accession numbers PRJNA760789.

## Abbreviations

8-OHDG: 8-Hydroxy-2′-deoxyguanosine
ALC: Ancestral-like coarse
BLAST: Basic local alignment search tool
DEGs: Differentially expressed genes
DEPs: Differentially expressed proteins
FISH: Fluorescence in situ hybridization
IG-DMR: Intergenic differentially methylated region
KO: Knockout
MF: Modern fine
MOET: Multiple ovulation and embryo transfer
OPLS-DA: Orthogonal partial least squares analysis
PCA: Principal component analysis
PRM: Parallel reaction monitoring
Q-PCR: Quantitative polymerase chain reaction
ROS: Reactive oxygen species
RT-PCR: Reverse transcription polymerase chain reaction
s-ITSs: Short interstitial telomeric sequences
SNP: Single nucleotide polymorphism
TEM: Transmission electron microscopy
TUNEL: Terminal deoxynucleotidyl transferase dUTP nick end labeling
WGBS: Whole genome bisulfite sequencing
