# Supplementary figures and images for: Epigenetic mechanism of Gtl2-miRNAs causes the primitive sheep characteristics found in purebred Merino sheep
Source: Cell Biosci. 2023 Oct 13;13:190. doi: 10.1186/s13578-023-01142-z (PMC10571318; doi:10.1186/s13578-023-01142-z)

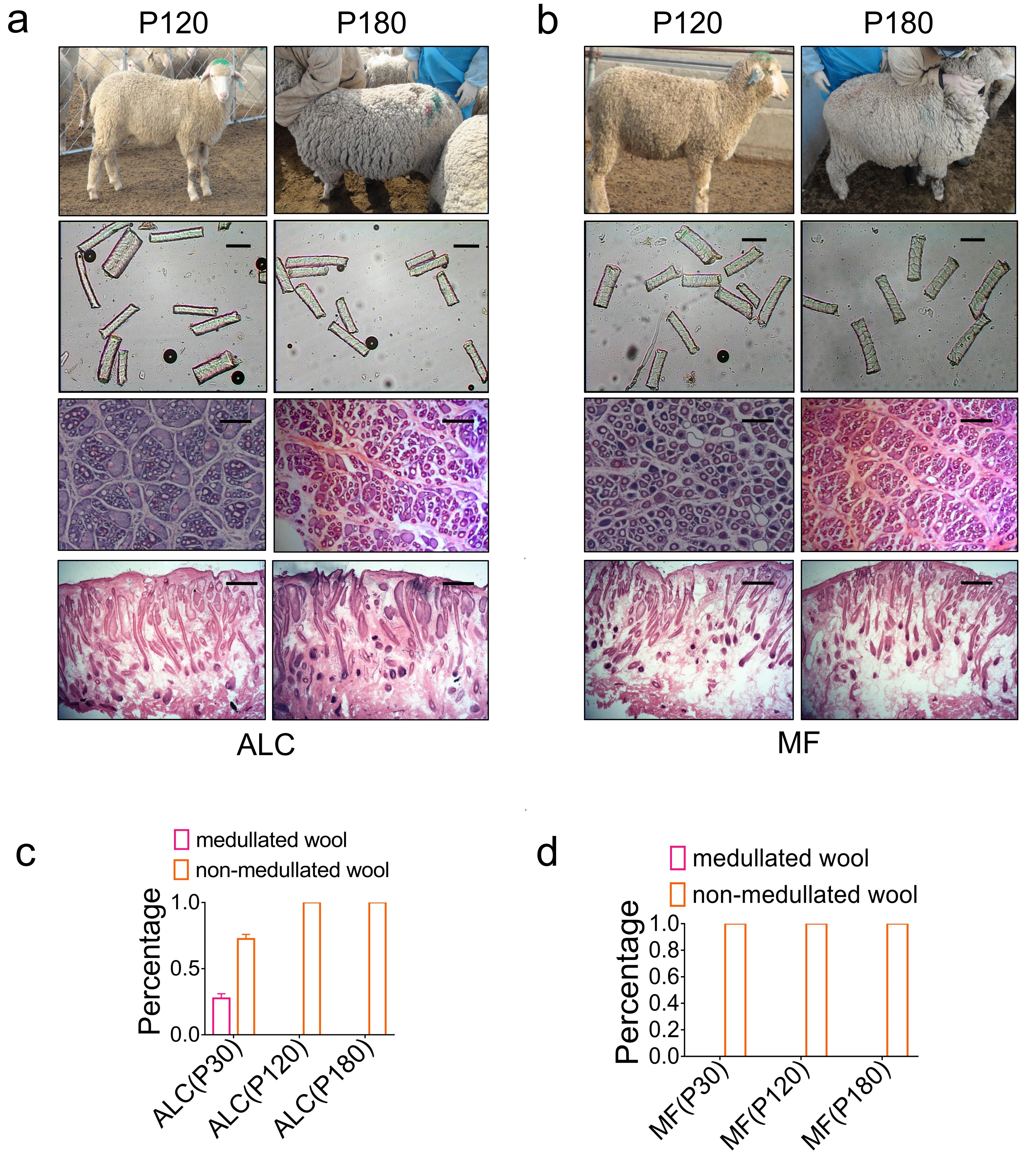

Supplement: Supplementary file 1 — Additional file 1: Figure S1. Wool characteristics of ancestral-like coarse (ALC) and modern fine (MF) wool sheep at P120 and P180. (a) The phenotypic properties of ALC wool sheep at P120 and P180. (b) The phenotypic properties of MF wool sheep at P120 and P180. (c) Proportion of medullated and unmedullated wool fibers at different developmental stages in ALC sheep. (d) Proportion of medullated and unmedullated wool fibers at different developmental stages in MF sheep. Figure S2. Wool characteristics of ancestral-like coarse (ALC) and modern fine (MF) wool sheep at P120 and P180. (a) Phylogenetic tree showing the evolutionary relationships of Gtl2-sITSs in various species. (b) Percentage of heterozygotes and homozygotes between ALC wool and MF wool varieties. (c) A small fraction of sITS in different species. Figure S3. Pathway enrichment analysis performed using the significantly downregulated metabolites in ancestral-like coarse (ALC) lambskin tissue. Figure S4. IRF2BP2 genotypes of ALC and MF wool lambs in reciprocal cross families. The primers were listed in Table S5. Figure S5. Embryonic weight of Meg3-IG-DMR-KO mice and their siblings (negative control), **P < 0.001. Figure S6. miRNAs in the Gtl2-miRNAs Locus inhibited multiple components of the PI3K-AKT Pathway. (a) The frequency of signaling pathways enriched by predicted target genes of up-regulated miRNAs in ALC group. (b) Schematic of the PI3K-mTOR pathway. (c) The up-regulated differentially expressed (DE) miRNAs at Gtl2-miRNAs locus and their predicted target genes in the PI3K-AKT pathway. Figure S7. Similar molecular mechanisms affect ALC wool traits and quality of lamb fur. a Curve graph of wool diameter distribution of Tan sheep. b Proportion of medullated and non-medullated wool of Tan sheep. c Integrated analysis of upregulated miRNAs and the functional annotation of their target genes between early developmental Tan and ALC lambs [38]. d Birth weight of ALC and MF wool lambs. e The skin ROS level [file 13578_2023_1142_MOESM1_ESM.zip › Figure S1.jpg]

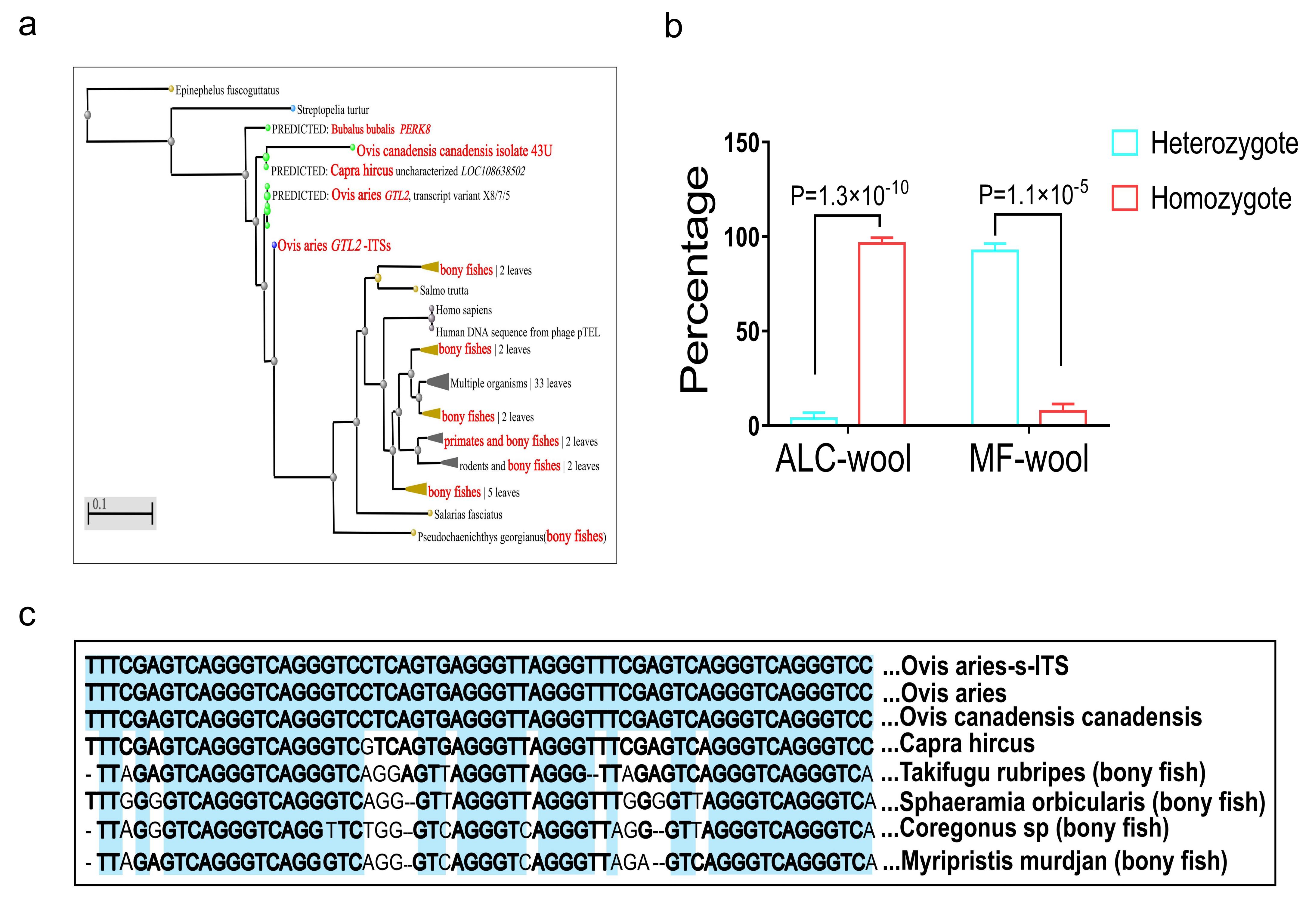

Supplement: Supplementary file 1 — Additional file 1: Figure S1. Wool characteristics of ancestral-like coarse (ALC) and modern fine (MF) wool sheep at P120 and P180. (a) The phenotypic properties of ALC wool sheep at P120 and P180. (b) The phenotypic properties of MF wool sheep at P120 and P180. (c) Proportion of medullated and unmedullated wool fibers at different developmental stages in ALC sheep. (d) Proportion of medullated and unmedullated wool fibers at different developmental stages in MF sheep. Figure S2. Wool characteristics of ancestral-like coarse (ALC) and modern fine (MF) wool sheep at P120 and P180. (a) Phylogenetic tree showing the evolutionary relationships of Gtl2-sITSs in various species. (b) Percentage of heterozygotes and homozygotes between ALC wool and MF wool varieties. (c) A small fraction of sITS in different species. Figure S3. Pathway enrichment analysis performed using the significantly downregulated metabolites in ancestral-like coarse (ALC) lambskin tissue. Figure S4. IRF2BP2 genotypes of ALC and MF wool lambs in reciprocal cross families. The primers were listed in Table S5. Figure S5. Embryonic weight of Meg3-IG-DMR-KO mice and their siblings (negative control), **P < 0.001. Figure S6. miRNAs in the Gtl2-miRNAs Locus inhibited multiple components of the PI3K-AKT Pathway. (a) The frequency of signaling pathways enriched by predicted target genes of up-regulated miRNAs in ALC group. (b) Schematic of the PI3K-mTOR pathway. (c) The up-regulated differentially expressed (DE) miRNAs at Gtl2-miRNAs locus and their predicted target genes in the PI3K-AKT pathway. Figure S7. Similar molecular mechanisms affect ALC wool traits and quality of lamb fur. a Curve graph of wool diameter distribution of Tan sheep. b Proportion of medullated and non-medullated wool of Tan sheep. c Integrated analysis of upregulated miRNAs and the functional annotation of their target genes between early developmental Tan and ALC lambs [38]. d Birth weight of ALC and MF wool lambs. e The skin ROS level [file 13578_2023_1142_MOESM1_ESM.zip › Figure S2.jpg]

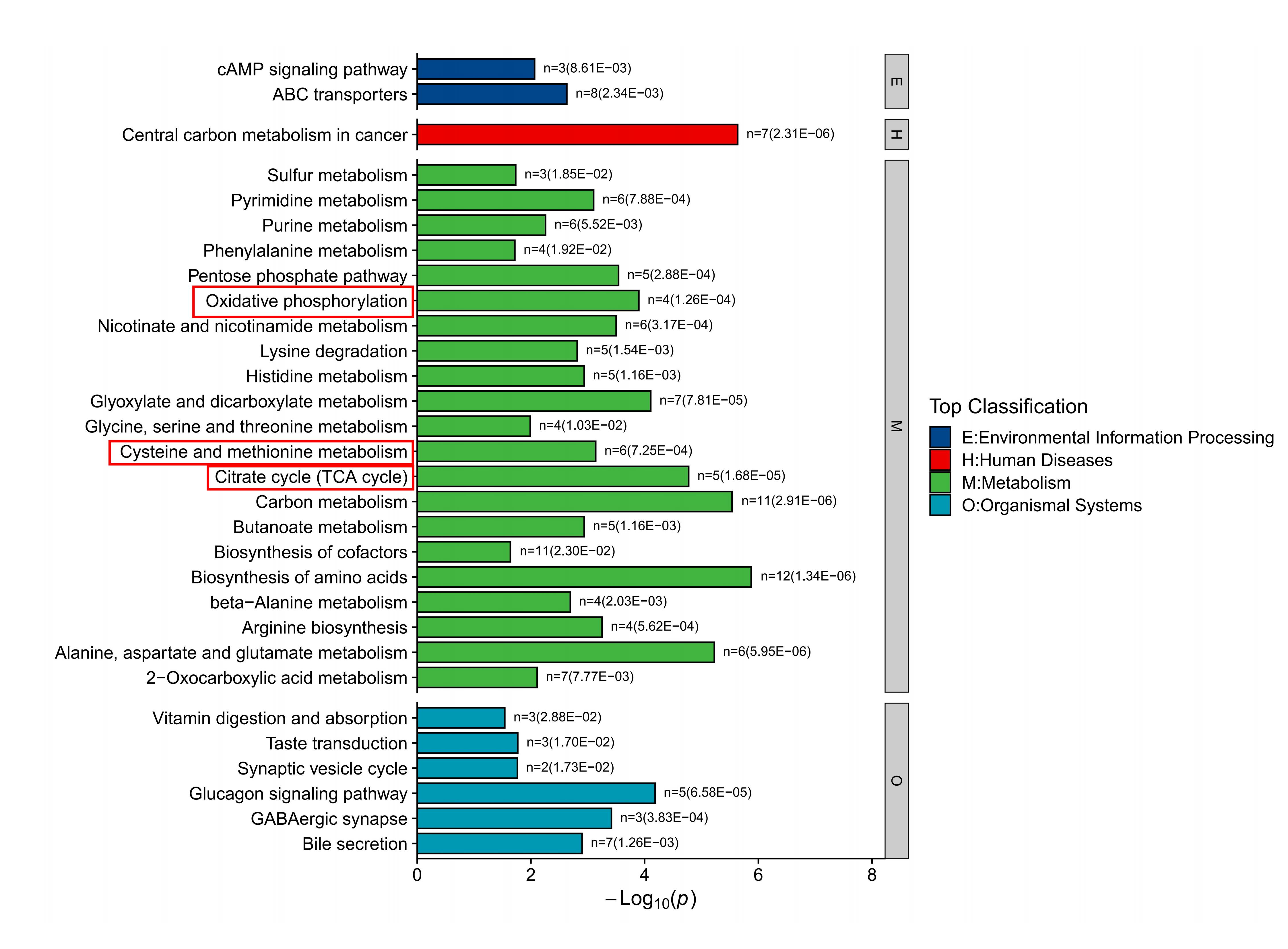

Supplement: Supplementary file 1 — Additional file 1: Figure S1. Wool characteristics of ancestral-like coarse (ALC) and modern fine (MF) wool sheep at P120 and P180. (a) The phenotypic properties of ALC wool sheep at P120 and P180. (b) The phenotypic properties of MF wool sheep at P120 and P180. (c) Proportion of medullated and unmedullated wool fibers at different developmental stages in ALC sheep. (d) Proportion of medullated and unmedullated wool fibers at different developmental stages in MF sheep. Figure S2. Wool characteristics of ancestral-like coarse (ALC) and modern fine (MF) wool sheep at P120 and P180. (a) Phylogenetic tree showing the evolutionary relationships of Gtl2-sITSs in various species. (b) Percentage of heterozygotes and homozygotes between ALC wool and MF wool varieties. (c) A small fraction of sITS in different species. Figure S3. Pathway enrichment analysis performed using the significantly downregulated metabolites in ancestral-like coarse (ALC) lambskin tissue. Figure S4. IRF2BP2 genotypes of ALC and MF wool lambs in reciprocal cross families. The primers were listed in Table S5. Figure S5. Embryonic weight of Meg3-IG-DMR-KO mice and their siblings (negative control), **P < 0.001. Figure S6. miRNAs in the Gtl2-miRNAs Locus inhibited multiple components of the PI3K-AKT Pathway. (a) The frequency of signaling pathways enriched by predicted target genes of up-regulated miRNAs in ALC group. (b) Schematic of the PI3K-mTOR pathway. (c) The up-regulated differentially expressed (DE) miRNAs at Gtl2-miRNAs locus and their predicted target genes in the PI3K-AKT pathway. Figure S7. Similar molecular mechanisms affect ALC wool traits and quality of lamb fur. a Curve graph of wool diameter distribution of Tan sheep. b Proportion of medullated and non-medullated wool of Tan sheep. c Integrated analysis of upregulated miRNAs and the functional annotation of their target genes between early developmental Tan and ALC lambs [38]. d Birth weight of ALC and MF wool lambs. e The skin ROS level [file 13578_2023_1142_MOESM1_ESM.zip › Figure S3.jpg]

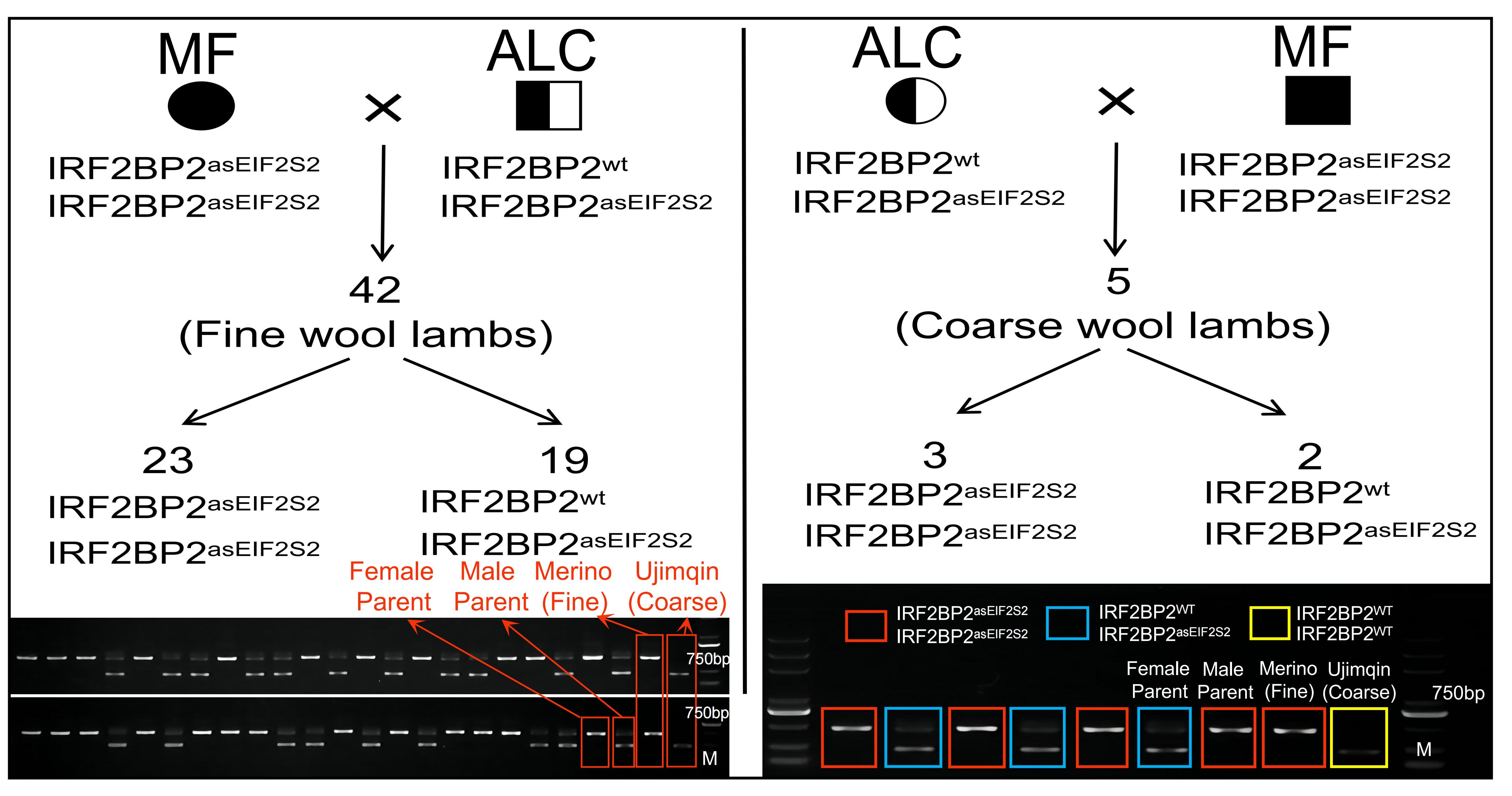

Supplement: Supplementary file 1 — Additional file 1: Figure S1. Wool characteristics of ancestral-like coarse (ALC) and modern fine (MF) wool sheep at P120 and P180. (a) The phenotypic properties of ALC wool sheep at P120 and P180. (b) The phenotypic properties of MF wool sheep at P120 and P180. (c) Proportion of medullated and unmedullated wool fibers at different developmental stages in ALC sheep. (d) Proportion of medullated and unmedullated wool fibers at different developmental stages in MF sheep. Figure S2. Wool characteristics of ancestral-like coarse (ALC) and modern fine (MF) wool sheep at P120 and P180. (a) Phylogenetic tree showing the evolutionary relationships of Gtl2-sITSs in various species. (b) Percentage of heterozygotes and homozygotes between ALC wool and MF wool varieties. (c) A small fraction of sITS in different species. Figure S3. Pathway enrichment analysis performed using the significantly downregulated metabolites in ancestral-like coarse (ALC) lambskin tissue. Figure S4. IRF2BP2 genotypes of ALC and MF wool lambs in reciprocal cross families. The primers were listed in Table S5. Figure S5. Embryonic weight of Meg3-IG-DMR-KO mice and their siblings (negative control), **P < 0.001. Figure S6. miRNAs in the Gtl2-miRNAs Locus inhibited multiple components of the PI3K-AKT Pathway. (a) The frequency of signaling pathways enriched by predicted target genes of up-regulated miRNAs in ALC group. (b) Schematic of the PI3K-mTOR pathway. (c) The up-regulated differentially expressed (DE) miRNAs at Gtl2-miRNAs locus and their predicted target genes in the PI3K-AKT pathway. Figure S7. Similar molecular mechanisms affect ALC wool traits and quality of lamb fur. a Curve graph of wool diameter distribution of Tan sheep. b Proportion of medullated and non-medullated wool of Tan sheep. c Integrated analysis of upregulated miRNAs and the functional annotation of their target genes between early developmental Tan and ALC lambs [38]. d Birth weight of ALC and MF wool lambs. e The skin ROS level [file 13578_2023_1142_MOESM1_ESM.zip › Figure S4.jpg]

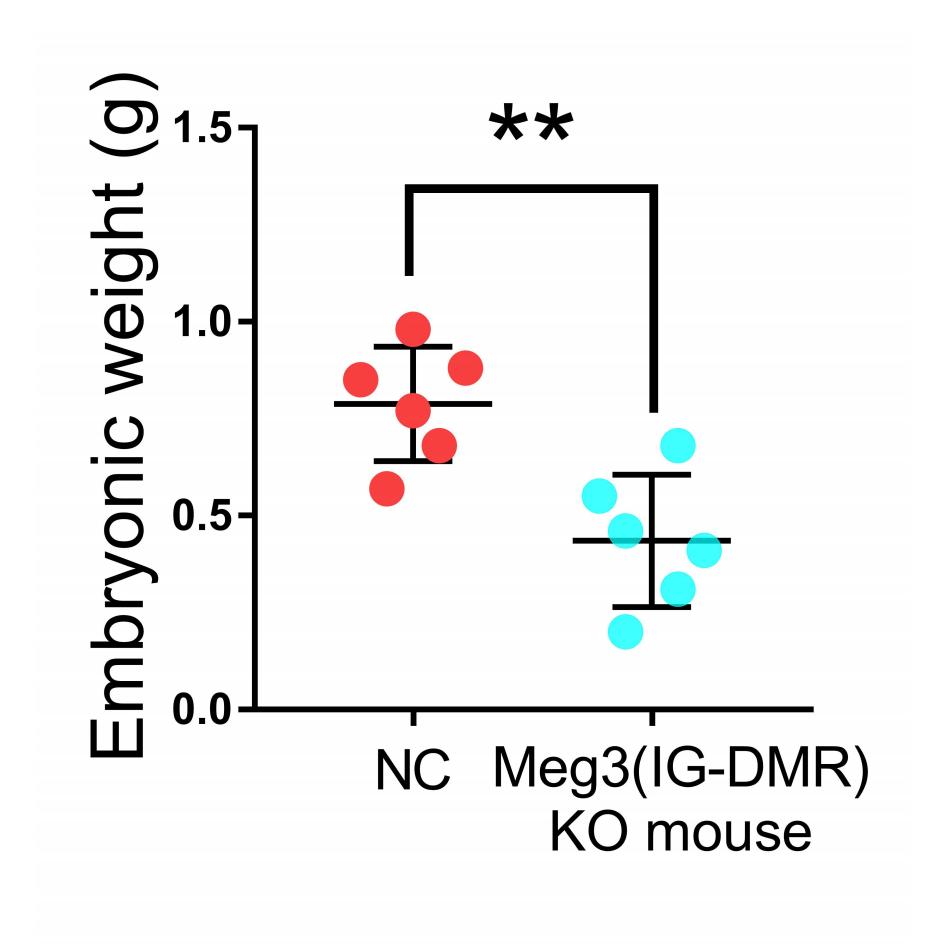

Supplement: Supplementary file 1 — Additional file 1: Figure S1. Wool characteristics of ancestral-like coarse (ALC) and modern fine (MF) wool sheep at P120 and P180. (a) The phenotypic properties of ALC wool sheep at P120 and P180. (b) The phenotypic properties of MF wool sheep at P120 and P180. (c) Proportion of medullated and unmedullated wool fibers at different developmental stages in ALC sheep. (d) Proportion of medullated and unmedullated wool fibers at different developmental stages in MF sheep. Figure S2. Wool characteristics of ancestral-like coarse (ALC) and modern fine (MF) wool sheep at P120 and P180. (a) Phylogenetic tree showing the evolutionary relationships of Gtl2-sITSs in various species. (b) Percentage of heterozygotes and homozygotes between ALC wool and MF wool varieties. (c) A small fraction of sITS in different species. Figure S3. Pathway enrichment analysis performed using the significantly downregulated metabolites in ancestral-like coarse (ALC) lambskin tissue. Figure S4. IRF2BP2 genotypes of ALC and MF wool lambs in reciprocal cross families. The primers were listed in Table S5. Figure S5. Embryonic weight of Meg3-IG-DMR-KO mice and their siblings (negative control), **P < 0.001. Figure S6. miRNAs in the Gtl2-miRNAs Locus inhibited multiple components of the PI3K-AKT Pathway. (a) The frequency of signaling pathways enriched by predicted target genes of up-regulated miRNAs in ALC group. (b) Schematic of the PI3K-mTOR pathway. (c) The up-regulated differentially expressed (DE) miRNAs at Gtl2-miRNAs locus and their predicted target genes in the PI3K-AKT pathway. Figure S7. Similar molecular mechanisms affect ALC wool traits and quality of lamb fur. a Curve graph of wool diameter distribution of Tan sheep. b Proportion of medullated and non-medullated wool of Tan sheep. c Integrated analysis of upregulated miRNAs and the functional annotation of their target genes between early developmental Tan and ALC lambs [38]. d Birth weight of ALC and MF wool lambs. e The skin ROS level [file 13578_2023_1142_MOESM1_ESM.zip › Figure S5.jpg]

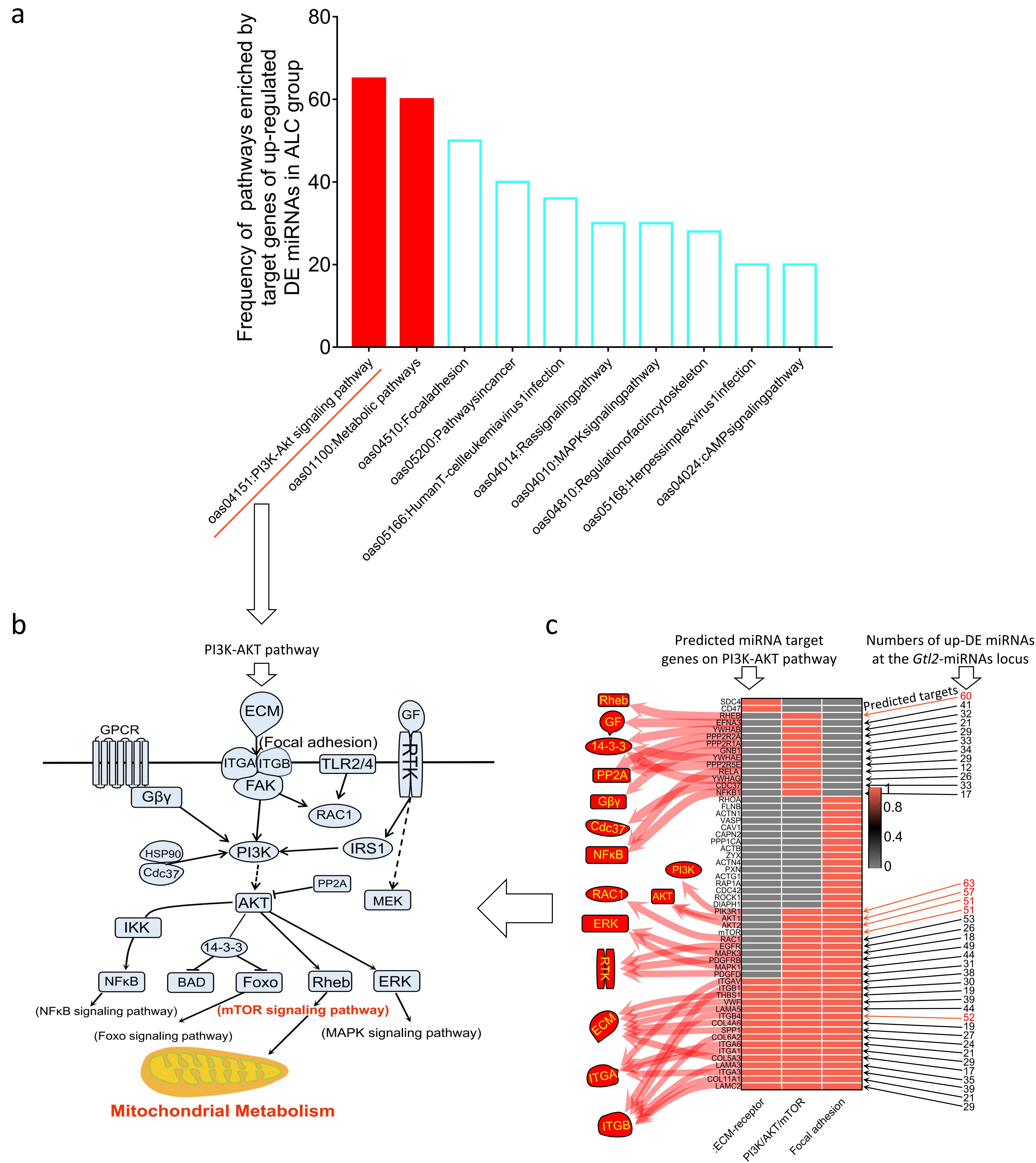

Supplement: Supplementary file 1 — Additional file 1: Figure S1. Wool characteristics of ancestral-like coarse (ALC) and modern fine (MF) wool sheep at P120 and P180. (a) The phenotypic properties of ALC wool sheep at P120 and P180. (b) The phenotypic properties of MF wool sheep at P120 and P180. (c) Proportion of medullated and unmedullated wool fibers at different developmental stages in ALC sheep. (d) Proportion of medullated and unmedullated wool fibers at different developmental stages in MF sheep. Figure S2. Wool characteristics of ancestral-like coarse (ALC) and modern fine (MF) wool sheep at P120 and P180. (a) Phylogenetic tree showing the evolutionary relationships of Gtl2-sITSs in various species. (b) Percentage of heterozygotes and homozygotes between ALC wool and MF wool varieties. (c) A small fraction of sITS in different species. Figure S3. Pathway enrichment analysis performed using the significantly downregulated metabolites in ancestral-like coarse (ALC) lambskin tissue. Figure S4. IRF2BP2 genotypes of ALC and MF wool lambs in reciprocal cross families. The primers were listed in Table S5. Figure S5. Embryonic weight of Meg3-IG-DMR-KO mice and their siblings (negative control), **P < 0.001. Figure S6. miRNAs in the Gtl2-miRNAs Locus inhibited multiple components of the PI3K-AKT Pathway. (a) The frequency of signaling pathways enriched by predicted target genes of up-regulated miRNAs in ALC group. (b) Schematic of the PI3K-mTOR pathway. (c) The up-regulated differentially expressed (DE) miRNAs at Gtl2-miRNAs locus and their predicted target genes in the PI3K-AKT pathway. Figure S7. Similar molecular mechanisms affect ALC wool traits and quality of lamb fur. a Curve graph of wool diameter distribution of Tan sheep. b Proportion of medullated and non-medullated wool of Tan sheep. c Integrated analysis of upregulated miRNAs and the functional annotation of their target genes between early developmental Tan and ALC lambs [38]. d Birth weight of ALC and MF wool lambs. e The skin ROS level [file 13578_2023_1142_MOESM1_ESM.zip › Figure S6.jpg]

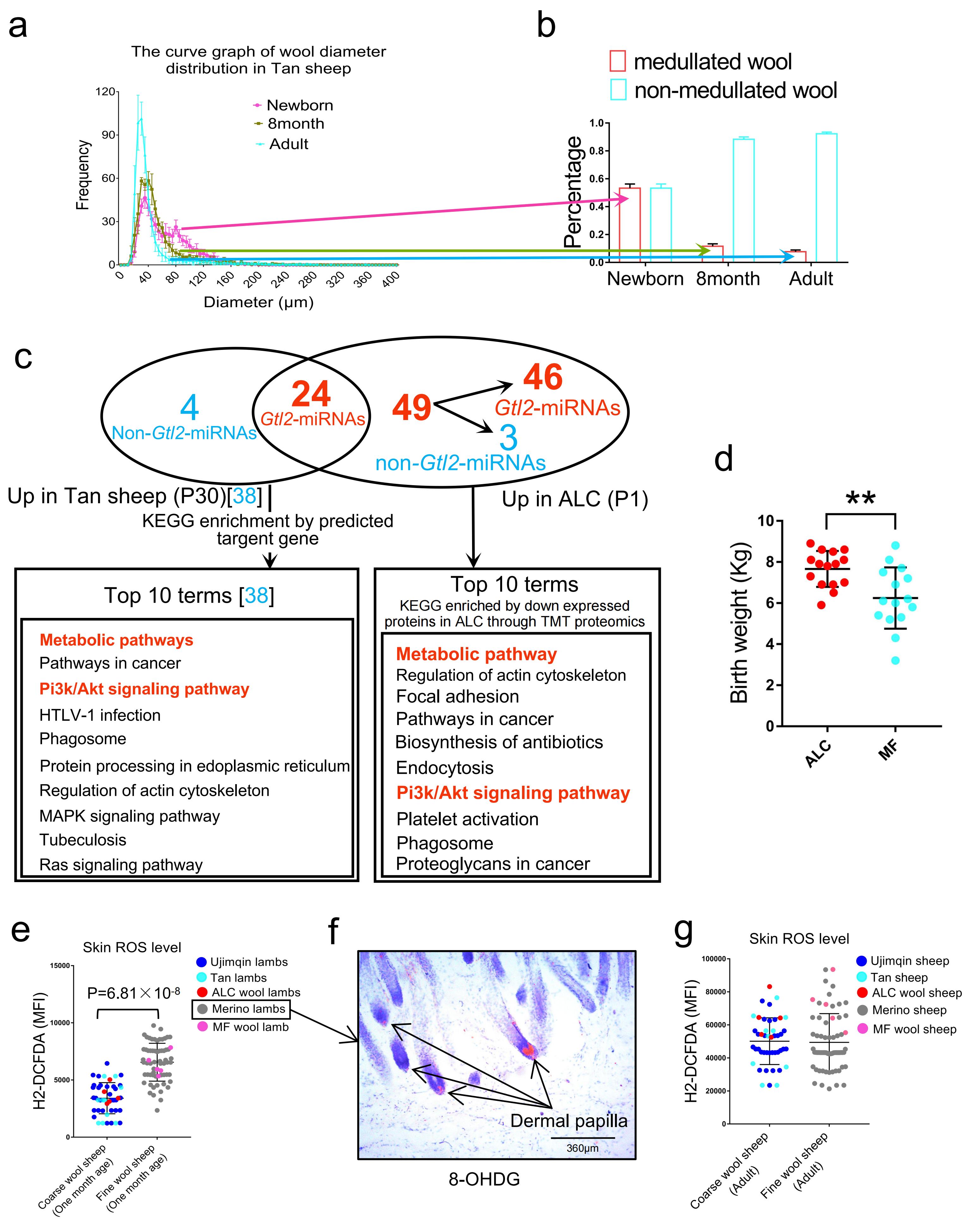

Supplement: Supplementary file 1 — Additional file 1: Figure S1. Wool characteristics of ancestral-like coarse (ALC) and modern fine (MF) wool sheep at P120 and P180. (a) The phenotypic properties of ALC wool sheep at P120 and P180. (b) The phenotypic properties of MF wool sheep at P120 and P180. (c) Proportion of medullated and unmedullated wool fibers at different developmental stages in ALC sheep. (d) Proportion of medullated and unmedullated wool fibers at different developmental stages in MF sheep. Figure S2. Wool characteristics of ancestral-like coarse (ALC) and modern fine (MF) wool sheep at P120 and P180. (a) Phylogenetic tree showing the evolutionary relationships of Gtl2-sITSs in various species. (b) Percentage of heterozygotes and homozygotes between ALC wool and MF wool varieties. (c) A small fraction of sITS in different species. Figure S3. Pathway enrichment analysis performed using the significantly downregulated metabolites in ancestral-like coarse (ALC) lambskin tissue. Figure S4. IRF2BP2 genotypes of ALC and MF wool lambs in reciprocal cross families. The primers were listed in Table S5. Figure S5. Embryonic weight of Meg3-IG-DMR-KO mice and their siblings (negative control), **P < 0.001. Figure S6. miRNAs in the Gtl2-miRNAs Locus inhibited multiple components of the PI3K-AKT Pathway. (a) The frequency of signaling pathways enriched by predicted target genes of up-regulated miRNAs in ALC group. (b) Schematic of the PI3K-mTOR pathway. (c) The up-regulated differentially expressed (DE) miRNAs at Gtl2-miRNAs locus and their predicted target genes in the PI3K-AKT pathway. Figure S7. Similar molecular mechanisms affect ALC wool traits and quality of lamb fur. a Curve graph of wool diameter distribution of Tan sheep. b Proportion of medullated and non-medullated wool of Tan sheep. c Integrated analysis of upregulated miRNAs and the functional annotation of their target genes between early developmental Tan and ALC lambs [38]. d Birth weight of ALC and MF wool lambs. e The skin ROS level [file 13578_2023_1142_MOESM1_ESM.zip › Figure S7.jpg]

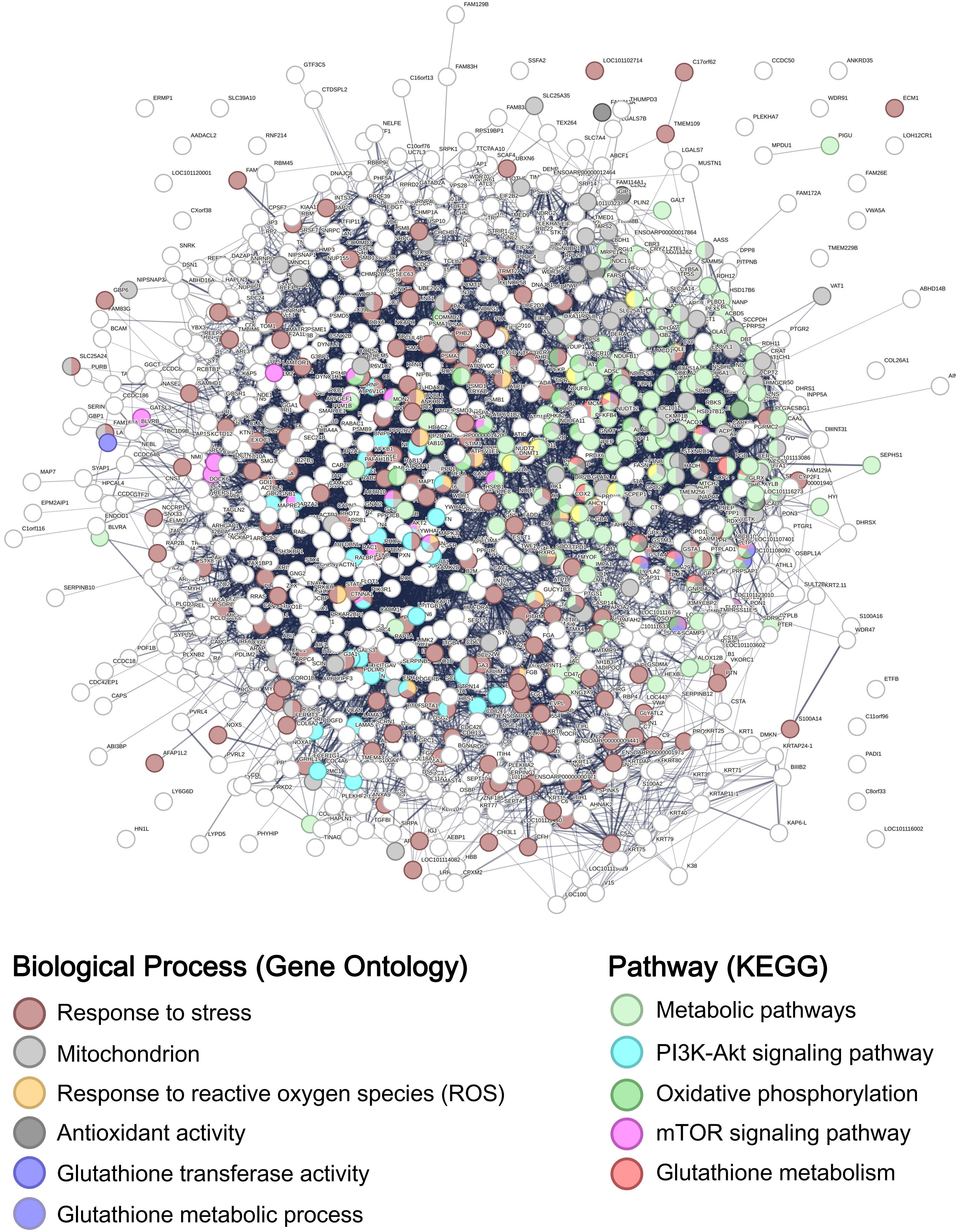

Supplement: Supplementary file 1 — Additional file 1: Figure S1. Wool characteristics of ancestral-like coarse (ALC) and modern fine (MF) wool sheep at P120 and P180. (a) The phenotypic properties of ALC wool sheep at P120 and P180. (b) The phenotypic properties of MF wool sheep at P120 and P180. (c) Proportion of medullated and unmedullated wool fibers at different developmental stages in ALC sheep. (d) Proportion of medullated and unmedullated wool fibers at different developmental stages in MF sheep. Figure S2. Wool characteristics of ancestral-like coarse (ALC) and modern fine (MF) wool sheep at P120 and P180. (a) Phylogenetic tree showing the evolutionary relationships of Gtl2-sITSs in various species. (b) Percentage of heterozygotes and homozygotes between ALC wool and MF wool varieties. (c) A small fraction of sITS in different species. Figure S3. Pathway enrichment analysis performed using the significantly downregulated metabolites in ancestral-like coarse (ALC) lambskin tissue. Figure S4. IRF2BP2 genotypes of ALC and MF wool lambs in reciprocal cross families. The primers were listed in Table S5. Figure S5. Embryonic weight of Meg3-IG-DMR-KO mice and their siblings (negative control), **P < 0.001. Figure S6. miRNAs in the Gtl2-miRNAs Locus inhibited multiple components of the PI3K-AKT Pathway. (a) The frequency of signaling pathways enriched by predicted target genes of up-regulated miRNAs in ALC group. (b) Schematic of the PI3K-mTOR pathway. (c) The up-regulated differentially expressed (DE) miRNAs at Gtl2-miRNAs locus and their predicted target genes in the PI3K-AKT pathway. Figure S7. Similar molecular mechanisms affect ALC wool traits and quality of lamb fur. a Curve graph of wool diameter distribution of Tan sheep. b Proportion of medullated and non-medullated wool of Tan sheep. c Integrated analysis of upregulated miRNAs and the functional annotation of their target genes between early developmental Tan and ALC lambs [38]. d Birth weight of ALC and MF wool lambs. e The skin ROS level [file 13578_2023_1142_MOESM1_ESM.zip › Figure S8.jpg]

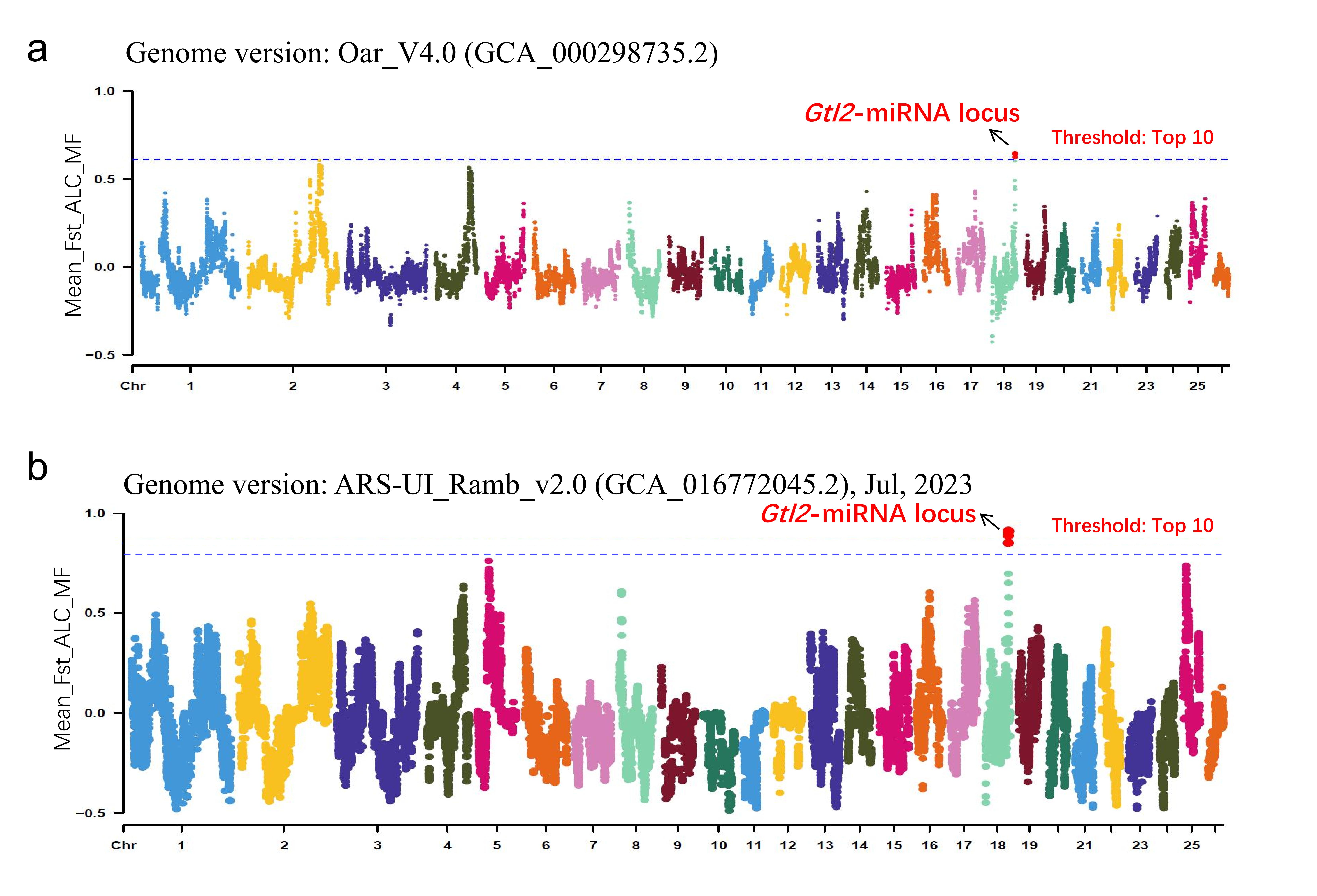

Supplement: Supplementary file 1 — Additional file 1: Figure S1. Wool characteristics of ancestral-like coarse (ALC) and modern fine (MF) wool sheep at P120 and P180. (a) The phenotypic properties of ALC wool sheep at P120 and P180. (b) The phenotypic properties of MF wool sheep at P120 and P180. (c) Proportion of medullated and unmedullated wool fibers at different developmental stages in ALC sheep. (d) Proportion of medullated and unmedullated wool fibers at different developmental stages in MF sheep. Figure S2. Wool characteristics of ancestral-like coarse (ALC) and modern fine (MF) wool sheep at P120 and P180. (a) Phylogenetic tree showing the evolutionary relationships of Gtl2-sITSs in various species. (b) Percentage of heterozygotes and homozygotes between ALC wool and MF wool varieties. (c) A small fraction of sITS in different species. Figure S3. Pathway enrichment analysis performed using the significantly downregulated metabolites in ancestral-like coarse (ALC) lambskin tissue. Figure S4. IRF2BP2 genotypes of ALC and MF wool lambs in reciprocal cross families. The primers were listed in Table S5. Figure S5. Embryonic weight of Meg3-IG-DMR-KO mice and their siblings (negative control), **P < 0.001. Figure S6. miRNAs in the Gtl2-miRNAs Locus inhibited multiple components of the PI3K-AKT Pathway. (a) The frequency of signaling pathways enriched by predicted target genes of up-regulated miRNAs in ALC group. (b) Schematic of the PI3K-mTOR pathway. (c) The up-regulated differentially expressed (DE) miRNAs at Gtl2-miRNAs locus and their predicted target genes in the PI3K-AKT pathway. Figure S7. Similar molecular mechanisms affect ALC wool traits and quality of lamb fur. a Curve graph of wool diameter distribution of Tan sheep. b Proportion of medullated and non-medullated wool of Tan sheep. c Integrated analysis of upregulated miRNAs and the functional annotation of their target genes between early developmental Tan and ALC lambs [38]. d Birth weight of ALC and MF wool lambs. e The skin ROS level [file 13578_2023_1142_MOESM1_ESM.zip › Figure S9.jpg]
